# *Streptomyces tardus* sp. nov.: A Slow-Growing Actinobacterium Producing Candicidin, Isolated From Sediments of the Trondheim Fjord

**DOI:** 10.3389/fmicb.2021.714233

**Published:** 2021-08-04

**Authors:** Stanislava Králová, Megan Sandoval-Powers, Dorelle V. Fawwal, Kristin F. Degnes, Anna Sofia Lewin, Geir Klinkenberg, Giang-Son Nguyen, Mark R. Liles, Alexander Wentzel

**Affiliations:** ^1^Department of Biological Sciences, Auburn University, Auburn, AL, United States; ^2^Department of Experimental Biology, Czech Collection of Microorganisms, Faculty of Science, Masaryk University, Brno, Czechia; ^3^Department of Biotechnology and Nanomedicine, SINTEF Industry, Trondheim, Norway

**Keywords:** systematics, *Streptomyces tardus* sp. nov., Actinobacteria, marine sediments, polyphasic taxonomy, biosynthetic potential

## Abstract

Marine environments are home to an extensive number of microorganisms, many of which remain unexplored for taxonomic novelty and functional capabilities. In this study, a slow-growing *Streptomyces* strain expressing unique genomic and phenotypic characteristics, P38-E01^*T*^, was described using a polyphasic taxonomic approach. This strain is part of a collection of over 8,000 marine Actinobacteria isolates collected in the Trondheim fjord of Norway by SINTEF Industry (Trondheim, Norway) and the Norwegian University of Science and Technology (NTNU, Trondheim, Norway). Strain P38-E01^*T*^ was isolated from the sediments of the Trondheim fjord, and phylogenetic analyses affiliated this strain with the genus *Streptomyces*, but it was not closely affiliated with other described species. The closest related type strains were *Streptomyces daliensis* YIM 31724^*T*^ (98.6%), *Streptomyces rimosus* subsp. *rimosus* ATCC 10970^*T*^ (98.4%), and *Streptomyces sclerotialus* NRRL ISP-5269^*T*^ (98.3%). Predominant fatty acids were C_16:0_ iso, C_16:0_, and Summed Feature 3, and the predominant respiratory quinones were MK-10(H_6_), MK-10(H_4_), and MK9(H_4_). The main polar lipids were identified as diphosphatidylglycerol, phosphatidylethanolamine, phosphatidylglycerol, and phosphoglycolipid. The whole-cell sugars were glucose, ribose, and in minor amounts, mannose. The cell wall peptidoglycan contained LL-diaminopimelic acid. The draft genome has a size of 6.16 Mb, with a %G + C content of 71.4% and is predicted to contain at least 19 biosynthetic gene clusters encoding diverse secondary metabolites. Strain P38-E01^*T*^ was found to inhibit the growth of the pathogenic yeast *Candida albicans* ATCC 90028 and a number of Gram-positive bacterial human and plant pathogens. Metabolites extracted from cultures of P38-E01^*T*^ were analyzed by mass spectrometry, and it was found that the isolate produced the antifungal compound candicidin. Phenotypic and chemotaxonomic signatures, along with phylogenetic analyses, distinguished isolate P38-E01^*T*^ from its closest neighbors; thus, this isolate represents a novel species of the genus *Streptomyces* for which the name *Streptomyces tardus* sp. nov. (P38-E01^*T*^ = CCM 9049^*T*^ = DSM 111582^*T*^) is proposed.

## Introduction

The genus *Streptomyces* was first proposed in the early 1940s (Waksman and Henrici, 1943) and has since become a popular subject of bioprospecting studies in search for new bioactive natural products. To date, the genus *Streptomyces* comprises a tremendous number of well-described species with vigorous ability to produce secondary metabolites that possess antibacterial (Waksman et al., 1944; Brown et al., 1976; Ser et al., 2016), antifungal (Law et al., 2017b), antioxidant (Tan et al., 2015; Law et al., 2017a), or anticancer activities (Tan et al., 2015). The broad range of biological activities of microbial secondary metabolites is the reason why *Streptomyces* spp. are comprehensively explored for novel taxa in order to uncover new drugs useful for therapeutic or preventative treatments. Streptomycetes are Gram-positive filamentous bacteria with three developmental stages of their life cycle, forming vegetative hyphae, reproductive aerial hyphae, and finally spores (Urem et al., 2016). Due to the high number of species validly described within the genus *Streptomyces* (over 800 to date) (Parte, 2018), characterization of any potentially novel species can pose a significant taxonomic challenge. Therefore, a polyphasic taxonomic approach starting with thorough biochemical and morphological testing supported by analyses of chemotaxonomic profiles and high-throughput molecular methods represents an appropriate and reliable way to characterize novel species within this genus (Ayed et al., 2018).

It is known that *Streptomyces* spp. are widely distributed in terrestrial ecosystems, especially soils. However, soil Actinobacteria have historically dominated the focus of novel taxa exploration and bioactivity screenings (Matsumoto and Takahashi, 2017). Thus, a current trend of studying Actinobacteria is focused on the isolation of new species from different biotopes such as mangroves (Law et al., 2019), lichens (Saeng-in et al., 2018), plant roots (Matsumoto and Takahashi, 2017; Ayed et al., 2018), or marine subaquatic areas (Dharmaraj, 2010; Shaik et al., 2017). In particular, the marine biosphere represents a large source of microbial diversity, and within this habitat, *Streptomyces* spp. are known to play a significant ecological role in organic matter turnover because of their extensive metabolic processes (Shaik et al., 2017). As such, these marine niches represent an underexplored habitat of novel *Streptomyces* and other Actinobacteria taxa with large genomic capacity to produce unique, bioactive compounds.

In the present study, a polyphasic taxonomic approach was applied in order to thoroughly characterize a slow-growing marine isolate, P38-E01^*T*^, affiliated with the genus *Streptomyces*. Based on the results obtained in this study, strain P38-E01^*T*^ (CCM 9049^*T*^ = DSM 111582^*T*^) isolated from sediments of a Norwegian fjord represents a novel species for which the name *Streptomyces tardus* sp. nov. is proposed. Antimicrobial assays indicated that strain P38-E01^*T*^ shows broad-spectrum activity against Gram-positive bacteria and the fungal strain *Candida albicans*, the latter of which was found to be linked to the production of the antifungal compound candicidin.

## Materials and Methods

### Isolation, Preservation, and Culture Conditions

Strain P38-E01^*T*^ was isolated in 2004 by the Gause Institute of New Antibiotics (Moscow, Russia) from marine sediments in the Trondheim fjord of Norway, harvested at 3- to 4-m depth between high and low tide (063°26′24.94″ N 010°20′50.28″ E). This work was part of an extensive marine Actinobacteria strain isolation campaign by SINTEF and the Norwegian University of Science and Technology NTNU (Trondheim, Norway). Details about the isolation methods from marine sediments used in this campaign are referred to in earlier studies (Bredholdt et al., 2007; Paulus et al., 2017).

Pure cultures were maintained on ISP 2 (International Streptomyces Project medium 2) (Shirling and Gottlieb, 1966) at room temperature for biochemical and morphological studies. For long-term preservation, a suspension of culture containing mycelia in ISP 2 supplemented with glycerol (20% v/v) was stored at −80°C. The reference strains used for comparative purposes, *Streptomyces daliensis* DSM 42095^*T*^, *Streptomyces rimosus* subsp. *rimosus* NRRL B-2659^*T*^, and *Streptomyces sclerotialus* NRRL B-2317^*T*^, were obtained from DSMZ (German Collection of Microorganisms and Cell Cultures, Braunschweig, Germany) and Agricultural Research Service (NRRL) Culture Collection (Peoria, IL, United States) and maintained under the same conditions as described above. Reference strains were tested in parallel with isolate P38-E01^*T*^.

### Morphology

Culture characteristics including colors of aerial and substrate mycelium of the strain P38-E01^*T*^ and reference strains following incubation at 30°C for 14 days were determined using various ISP media, namely, ISP 2 (yeast extract–malt agar), ISP 3 (oatmeal agar), ISP 4 (inorganic salts–starch agar), ISP 5 (glycerol–asparagine agar), ISP 6 (peptone–yeast extract iron agar), and ISP 7 (tyrosine agar), all prepared according to the method of Shirling and Gottlieb (1966). Production of melanin was observed on ISP 6 and ISP 7 media. Additionally, nutrient agar, Czapek–Dox agar (Atlas, 2004), and modified Bennett’s agar (Jones, 1949) were also used in order to describe macroscopic morphology. The color of colonies, reversed-phase colony color, and soluble pigments were determined using the NBS/ISCC color charts (Kelly et al., 1965). Scanning electron microscopy (Zeiss, Oberkochen, Germany) was used to observe microscopic morphology of strain P38-E01^*T*^, cultivated for 14 days on ISP 2 medium at 30°C (Supplementary Figure 1).

### Growth Conditions

Strain P38-E01^*T*^ was tested along with the reference strains for tolerance to different temperatures (3, 6, 8, 10, 15, 20, 25, 28, 30, 32, 35, 37, and 40°C) on ISP 2 medium. Tolerance to salinity was determined on ISP 2 medium with 0–6% (w/v) NaCl (at intervals of 0.5% w/v) at 30°C. Additionally, growth at different pH was tested on ISP 2 medium with pH ranging from 3 to 14 (at intervals of 0.5 pH unit) using the buffer system described by Barbour et al. (2017). The growth was observed periodically for up to 4 weeks.

### Biochemical and Physiological Characteristics

Catalase and oxidase activity were evaluated as described by Smibert and Krieg (Kamlage, 1996). Urease activity was evaluated on Christensen’s medium (Christensen, 1946), and nitrate reduction was tested using ISP media according to the method by Gordon et al. (1974). Other relevant tests included DNA hydrolysis test (DNAse agar, BD Biosciences), test for hydrolysis of gelatin and Tween 80 (Páčová and Kocur, 1984), tests for hydrolysis of starch and esculin (Barrow and Feltham, 1993), tests for hydrolysis of casein and tyrosine (Kurup and Babcock, 1979), and tests for hydrolysis of xanthin and hypoxanthine (*Nocardia* hydrolysis kit, Hardy Diagnostics). Cellulolytic activity was evaluated using cellulose (ISP 2 broth with strip of Whatman paper No. 1), and growth and/or degradation activity were observed periodically for 4 weeks. Utilization of different carbon sources was evaluated according to Shirling and Gottlieb’s protocol (Shirling and Gottlieb, 1966) using ISP 9 medium supplemented with 1% (w/v) carbon source and cultivated in two parallels. Additionally, enzymatic activity was evaluated using an API ZYM kit (bioMérieux, France) according to manufacturer’s instructions.

### Antibiotic Susceptibility Testing

For antibiotic resistance profiling the Kirby–Bauer disc-diffusion method on Mueller–Hinton agar was used (Bauer et al., 1966). Antibiotic discs used for these purposes were ampicillin (10 μg), chloramphenicol (30 μg), gentamicin (10 μg), kanamycin (30 μg), neomycin (5 μg), streptomycin (10 μg), tetracycline (30 μg), penicillin G (10 μg), clindamycin (2 μg), cefixime (5 μg), ciprofloxacin (5 μg), erythromycin (15 μg), and polymyxin B (300 U). The Clinical and Laboratory Standards Institute (CLSI) standards were strictly followed for cultivation and inhibition zone diameter reading (CLSI, 2015). Strain P38-E01^*T*^ was susceptible to all antibiotics used, except for chloramphenicol (30 μg), penicillin G (10 μg), and cefixime (5 μg).

### Analysis of Fatty Acid Methyl Esters

Cells for the fatty acid methyl ester (FAME) analysis were grown in trypticase soy broth (BBL). The media for FAME were prepared by dispensing 20 ml of TSB into 150-ml Erlenmeyer flasks, sterilized by autoclaving (30 min/121°C). All strains were cultivated in parallel for 10 days at 28°C on a platform shaker at 150 rpm. Cells were harvested by filtration; followed by the extraction procedure described in a standard protocol by Sasser (1990). Analysis of FAMEs was performed using an Agilent 7890B gas chromatograph according to the standard protocol of the Sherlock MIDI Identification System (MIDI Sherlock version 6.2, MIDI database RTSBA 6.21).

### Analysis of Peptidoglycan, Quinones, and Polar Lipids

Analyses of peptidoglycan, respiratory quinones, and polar lipids were carried out by the Identification Service, Leibniz-Institut DSMZ–Deutsche Sammlung von Mikroorganismen und Zellkulturen GmbH (Braunschweig, Germany). Cell mass was hydrolyzed and the hydrolysates analyzed according to protocols 1 and 3 of Schumann (2011) for whole-cell sugars and diaminopimelic acid. Menaquinones and polar lipids were extracted from freeze-dried cell material in a two-stage method to first extract the respiratory quinones followed by the polar lipids (Tindall, 1990a, b). Menaquinones were then analyzed by HPLC-DAD-MS/MS. Polar lipids were extracted using a choroform:methanol:0.3% aqueous NaCl mixture and recovered into the chloroform phase, modified after Bligh and Dyer (1959). Separation was done by two-dimensional silica gel thin-layer chromatography with the first direction developed in chloroform:methanol:water and the second in chloroform:methanol:acetic acid:water. Total lipid material was detected using molybdatophosphoric acid, and specific functional groups were detected using spray reagents specific for defined functional groups (Tindall et al., 2007).

### Genomic and Phylogenetic Analyses

The draft genome sequence of strain P38-E01^*T*^ was determined using a combination of Illumina HiSeq and Pacific Biosciences (PacBio) sequencing. Extraction of genomic DNA from strain P38-E01^*T*^ and both Illumina short-read and PacBio long-read sequencing were performed by BaseClear BV (Leiden, Netherlands). For Illumina short-read sequencing, a paired-end library was generated using genomic DNA of strain P38-E01^*T*^ and approximately 300 bp of tagmentation using the Nextera XT DNA Library Preparation Kit (Illumina) according to the manufacturer instructions of the manufacturer. A total of 2,157,583 2 × 125-bp paired-end sequence reads were generated using the Illumina HiSeq2500 system, yielding 516 Mb sequence data. FASTQ sequence files were generated using the Illumina Casava pipeline version 1.8.3. Initial quality assessment was based on data passing the Illumina Chastity filtering. Subsequently, reads containing PhiX control signal were removed by filtering. In addition, reads containing (partial) adapters were clipped (up to a minimum read length of 50 bp). Quality assessment was based on the remaining reads using the FASTQC quality control tool version 0.10.0. The average quality score (Phred) obtained was 38.27. The quality of the Illumina reads was improved further by trimming low-quality bases using BBDuk, which is a part of the BBMap suite version 36.77.^1^ High-quality reads were assembled into contigs using ABySS version 2.0 (Jackman et al., 2017), also incorporating paired-end read For the PacBio long-read sequencing; a total of 579,093 reads (mean read length 4,440 bases) were generated using the PacBio Sequel instrument, yielding 2,571 Mb of sequence data. The data collected were processed and filtered using the SMRT Link software suite, and subreads shorter than 50 bp were discarded. For scaffolding, the long reads were mapped to the Illumina draft assembly using BLASR version 1.3.1 (Chaisson and Tesler, 2012). Based on these alignments, the contigs were linked together and placed into scaffolds. The orientation, order, and distance between the contigs were estimated using SSPACE-LongRead version 1.0 (Boetzer and Pirovano, 2014). Using Illumina reads, gapped regions within scaffolds were (partially) closed using GapFiller version 1.10 (Boetzer and Pirovano, 2012). Finally, assembly errors and the nucleotide disagreements between the Illumina reads and scaffold sequences were corrected using Pilon version 1.10 (Walker et al., 2014). By that means, a draft genome sequence in 10 scaffolds was obtained, which has been deposited at NCBI GenBank under accession number JAELVF000000000, and the version described in this paper is version JAELVF020000000. The quality of the assembly calculated by Quast version 5.1 is listed in Table 3 (Gurevich et al., 2013). For whole-genome analyses, *S. daliensis* DSM 42095^*T*^ was sequenced and its genome assembled as described above, with the sequence deposited at NCBI GenBank under accession number JAGSMN000000000. Whole-genome sequences of *S. rimosus* subsp. *rimosus* ATCC 10970^*T*^ and *S. sclerotialus* NRRL ISP-5269^*T*^, *S. harbinensis* CGMCC 47047^*T*^, *S. alkaliterrae* OF1^*T*^, and *S. durbertensis* DSM 104538^*T*^ were retrieved as genome assemblies from the NCBI GenBank database.

The 16S rRNA gene sequence of strain P38-E01^*T*^ (1,527 bp) was extracted from the draft genome sequence data and initially analyzed using the EzBioCloud server (Yoon et al., 2017). For phylogenetic analysis, the 16S rRNA gene sequences of the type strains of closely related *Streptomyces* species were retrieved from NCBI GenBank database. Sequences were aligned using the ClustalW algorithm, and phylogenetic analysis was performed using the MEGA 10.0.5 software (Kumar et al., 2018). Phylogenetic trees were constructed using the neighbor-joining (Saitou and Nei, 1987), maximum-likelihood (Felsenstein, 1981), and maximum-parsimony (Kluge and Farris, 1969) methods, evaluated by bootstrap analysis with 1,000 replications (Felsenstein, 1985). For greater taxonomic resolution, a multi-locus sequence analysis (MLSA) was performed by uploading the draft genome assemblies of strain P38-E01^*T*^ and the closely related type strains to the Automated Multi-Locus Species Tree (autoMLST) web tool (Alanjary et al., 2019). A concatenated alignment was generated in autoMLST using default parameters with 100 core genes from 50 closely related genomes, and an IQ-TREE ultrafast bootstrap analysis (Nguyen et al., 2015) with 1,000 replications was performed. A phylogenomic tree was calculated using the Type Strain Genome Software (TYGS) implanting the Genome BLAST Distance Phylogeny (GBDP) strategy (Meier-Kolthoff and Göker, 2019). Calculation of orthoANI values (orthologous average nucleotide identity) and generation of an ANI heat map was performed by OAT software version 0.93.1 (Lee et al., 2016). Digital DNA–DNA hybridization (dDDH) values were generated in order to confirm genomic uniqueness of strain P38-E01^*T*^. All genomes were subjected to the Genome to Genome Distance Calculator (GGDC) version 2.1 (Meier-Kolthoff et al., 2013).

### Bioinformatic Analysis

Genome annotation performed by the NCBI Prokaryotic Annotation Pipeline (Haft et al., 2018) and the eggNOG-mapper (Huerta-Cepas et al., 2019) was used for the prediction and classification of functional groups. Carbohydrate Active Enzymes (CAZymes) were predicted using the dbCAN2 meta server (Zhang et al., 2018). PlasmidFinder v2.1 (Carattoli et al., 2014) was used for identification of plasmid, and the presence of putative prophage regions was analyzed by PHASTER (Arndt et al., 2016) and Prophage Hunter (Song et al., 2019) tools. The *in silico* resistome of strain P38-E01^*T*^ was predicted using the Resistance Genes Identifier (RGI, v5.1.1) with The Comprehensive Antibiotic Resistance Database (CARD, v3.1.1) (Alcock et al., 2020). CRISPRDetect v2.3 (Biswas et al., 2016) was applied for the detection of CRISPR (clustered regularly interspaced short palindromic repeats) arrays. AntiSMASH version 6.0.0 (Blin et al., 2021) was used to detect presence of biosynthetic gene clusters (BGCs) predicted to produce secondary metabolites.

### Antimicrobial Activity

For initial assessments of isolate P38-E01^*T*^ ability to produce antimicrobial metabolites, supernatants from a broth culture were screened for the ability to inhibit the growth of various bacterial or fungal strains by a spot-on-lawn assay. Target organisms for antimicrobial activity assays included *Micrococcus luteus* ATCC 10240, *Staphylococcus aureus* (MRSA) Xen29, MRSA strain EAMC30, *C. albicans* ATCC 90028, *Escherichia coli* BL21, *Clavibacter michiganensis* subsp. *michiganensis* 89C-4, and *Curtobacterium flaccumfaciens* subsp. *flaccumfaciens* CV3. Strain P38-E01^*T*^ was grown in shake flasks with stainless steel coiled spring containing 100 ml each of six different media types of yeast extract–malt extract (YEME) broth with and without sucrose (Shepherd et al., 2010), marine fermentation (MF) broth (Costa et al., 2017), glucose yeast extract (GYE) broth (Koo et al., 1991), ISP 2 broth with and without artificial seawater, and TSB for 35 days at 25°C and shaking at 200 rpm. At 7-day intervals, a 1-ml volume was pulled from each culture, cells were pelleted by centrifugation at 4,000 × *g*, and supernatants were filtered through a 0.2-μm microporous membrane. Target organisms were swabbed on the surface of a tryptic soy agar (TSA) plate, after which, 10 μl of cell-free supernatant was spotted on the surface of the agar. A 10-μl spot of each media type was used as a control. Plates were incubated for 24–48 h at the appropriate temperature for the test organism, and the antimicrobial activity was determined by the presence or absence of a zone of inhibition (ZOI) surrounding the test samples.

### Identification of Bioactive Compounds

To identify the compounds responsible for the observed bioactivity, extracts of strain P38-E01^*T*^ were fractionated and analyzed by LC-MS. Strain P38-E01^*T*^ was grown in three media types all containing 0.5 × artificial sea water, i.e., 5010SW (15 g/L of sucrose, 1 g/L of NaNO_3_, 0.5 g/L of KH_2_PO_4_, 0.25 g/L of MgSO_4_ × 7H_2_O, 0.25 g/L of KCl, 0.005 g/L of FeSO_4_ × 7H_2_O), 5334SW (10 g/L of glucose, 5 g/L of soy meal, 0.1 g/L of CaCO_3_, 0.0005 g/L of CoCl_2_ × 2H_2_O), and 5254SW (7.5 g/L of glucose, 7.5 g/L of soy meal, 2.5 g/L of corn steep, 1.0 g/L of CaCO_3_, 2.5 g/L of NaCl), in 2-ml deep-well plates (Greiner Bio One) with 600 μl of culture/well. To limit pellet formation, 3-mm-diameter glass beads were added to each well with one glass bead per well. The cultures were incubated at 28°C and 800 rpm. The 5010SW cultures were incubated for 13 days, and the 5334SW and 5254SW cultures were incubated for 7 days. The cultures were dried by lyophilization and then extracted with 1× culture volume DMSO for 1 h. Cell-free extracts were tested for activity against *Enterococcus faecium* CCUG 37832, *Kocuria rhizophila* ATCC 9341, *Pseudomonas aeruginosa* ATCC 15692, and *C. albicans* CCUG 39333. The assay was performed in 384-well plates, and a Tecan EVO liquid-handling workstation was used for robotic dilution, sample preparation, and inoculation. Gram-negative indicator organisms were cultivated in 0.5 × Muller–Hinton broth 2 medium and Gram positive in 1× Brain heart infusion medium. Cultivation volume in each well was 40 μl with 3.15% DMSO extract. Each DMSO extract was assayed undiluted, 4× and 16× diluted. The assay plates were incubated for 10 h without shaking. Growth was measured as OD_600_, and 100% growth was defined as OD_600_ of cultures to which was added 3.15% DMSO as a negative control. For fractionation, an Agilent 1260 HPLC equipped with a diode array detector (DAD) and an Agilent Zorbax Bonus-RP, 4.6 × 75 mm, 1.8-μm column was used. The mobile phase consisted of acetonitrile and 10 mM ammonium acetate with a flow rate of 0.9 ml/min. The acetonitrile gradient was linearly increased from 5 to 95% for 24 min, and fractions were sampled every minute for the run. Fractions from two parallel runs were collected in 96 deep-well plates. The resulting fractions were assayed for antifungal activity against *C. albicans* CCUG 39333, *M. luteus* ATCC9341, and *E. faecium* CCUG 37832. The DMSO crude extracts of strain P38-E01^*T*^ were analyzed on an Agilent 1290 HPLC system with a Zorbax bonus RP, 2.1 × 50 mm, 3.5-μm column connected to a DAD, and a QTOF was used to precisely identify antifungal metabolites.

## Results and Discussion

### Phenotypic Characterization of Strain P38-E01^*T*^

In general, strain P38-E01^*T*^ grew well on the media types tested except for ISP 3 and Czapek–Dox agar, and the observed morphological features were aligned with the characteristics typical of the genus *Streptomyces*. Strain P38-E01^*T*^ produced white aerial mycelia on all tested media with the exception of ISP2 medium, on which it produced pale yellow aerial mycelia. The color of substrate mycelia differed on various media from white to yellow. Colony morphology captured by scanning electron microscopy is shown in Supplementary Figure 1. No melanin production was observed when strain P38-E01^*T*^ was grown on ISP 6 or ISP 7. In comparison, the reference strains grew on all media types tested and showed melanin pigment production on ISP 6 and/or ISP 7. The growth and cultural characteristics of strain P38-E01^*T*^ in comparison with the reference strains are described in Supplementary Table 1.

The temperature range for the growth of strain P38-E01^*T*^ was 28–32°C with an optimum at 30°C, a narrower range of temperature tolerance than that observed for the reference strains (Table 1). The highest tolerated concentration of NaCl was 5.0%, and strain P38-E01^*T*^ was able to grow in a pH range of 6.5–11.0. Strain P38-E01^*T*^ showed different biochemical and physiological characteristics in at least 26 of the physiological and biochemical features tested in comparison with one or more of the closest related reference strains. Notably, strain P38-E01^*T*^ is specific by its hydrolytic activities lacking only the ability to hydrolyze esculin. It was observed to express less enzymatic activities compared with *S. daliensis* DSM 42095^*T*^ and *S. rimosus* subsp. *rimosus* NRRL B-2659^*T*^ but seems to be more active than *S. sclerotialus* NRRL B-2317^*T*^ using API ZYM. All distinguishable traits are listed in Table 1.

**TABLE 1:** Phenotypic characteristics that differentiate *Streptomyces tardus* sp. nov. from closely related *Streptomyces* species.

Test	1.	2.	3.	4.
Temperature range	28–32°C	15–40°C	13–45°C	15–45°C
Growth in 5% NaCl	+	+	+	−
Growth in 6% NaCl	−	+	+	−
pH range	6.5–11	4.5–12.0	4.0–13	5.0–12.0
Nitrate reduction	−	−	+	+
Urease production	−	+	+	−
**Hydrolysis of:**				
Esculine	−	+	w	+
Hypoxanthin	+	−	+	+
Tween 80	+	−	+	−
Utilization of cellulose	+	+	−	+
Xanthin	+	−	+	+
**Carbon sources:**				
D-lactose	−	+	+	+
D-sorbitol	−	−	+	+
D-xylose	+	+	−	+
L-rhamnose	−	+	−	+
*myo*-inositol	−	−	+	+
Raffinose	−	−	+	−
Salicin	−	−	−	+
Sucrose	−	−	−	+
**API ZYM**				
Alkaline phosphatase	+	+	+	−
Acid phosphatase	−	+	+	w
α-chymotrypsin	−	−	−	−
α-glucosidase	−	+	+	−
α-mannosidase	−	−	w	−
β-galactosidase	−	+	+	−
β-glucosidase	−	+	w	−
Cystine arylamidase	−	w	+	−
Lipase (C 14)	+	−	−	−
Trypsin	−	−	w	−
Valine arylamidase	−	w	+	−

*Strains: 1, strain P38-E01^*T*^; 2, *S. daliensis* DSM 42095^*T*^; 3, *S. rimosus* subsp. *rimosus* NRRL B-2659^*T*^; 4, *S. sclerotialus* NRRL B-2317^*T*^.*

### Chemotaxonomic Analysis of Strain P38-E01^*T*^

**FIGURE 1:**
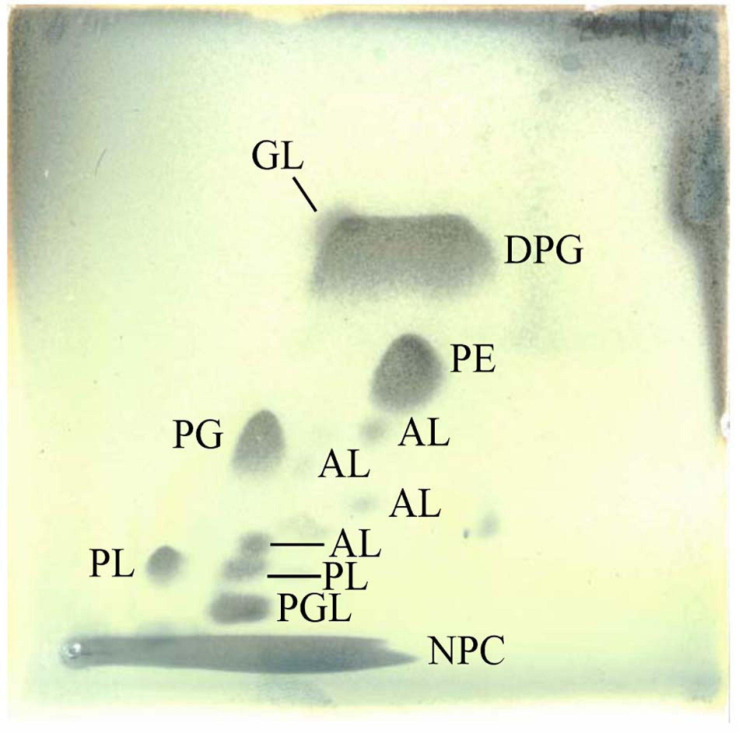
Polar lipids of strain P38-E01^*T*^. Total lipids were visualized by two-dimensional TLC applying 5% molybdatophosphoric acid. Abbreviations: AL, aminolipid; DPG, diphosphatidylglycerol; GL, glycolipid; NPC, non-polar compound (co-extracted with polar lipids); PE, phosphatidylethanolamine; PG, phosphatidylglycerol; PGL, phosphoglycolipid; PL, phospholipid.

Analysis of fatty acids showed that the predominant fatty acids of strain P38-E01^*T*^ were iso-C_16:0_ (30.0%), C_16:0_ (9.1%), and Summed Feature 3 (C_16:1_ *ω7c*/C_16:1_ *ω6c*) (8.9%). The fatty acid profile revealed that strain P38-E01^*T*^ produces major amounts of iso- and anteiso-, branched fatty acids, which are in concordance with the characteristics of the genus *Streptomyces* (Kämpfer, 2015). The closest relatives, *S. daliensis* DSM 42095^*T*^, *S. rimosus* subsp. *rimosus* NRRL B-2659^*T*^, and *S. sclerotialus* NRRL B-2317^*T*^ all were distinguishable from strain P38-E01^*T*^ by higher amounts of anteiso-C_15:0_ and anteiso-C_17:0_, and lower/trace amounts of iso-C_16:0_, C_17:0_, and Summed Feature 3. Comparison of fatty acid profiles is listed in Table 2. The predominant respiratory quinones were MK-10(H_6_), MK-10(H_4_), and MK9(H_4_). The main polar lipids were identified as diphosphatidylglycerol, phosphatidylethanolamine, phosphatidylglycerol, and phosphoglycolipid (Figure 1). The whole-cell sugars were glucose, ribose, and in minor amounts, mannose. The cell wall peptidoglycan contains LL-diaminopimelic acid. All described chemotaxonomic characteristics are typical for the genus *Streptomyces* (Kämpfer, 2015) and confirmed that the analyzed strain does belongs to this genus.

**TABLE 2:** Cellular fatty acid composition (%) of strain P38-E01^*T*^ and closely related *Streptomyces* spp.

Fatty acid	1.	2.	3.	4.
iso-C_14:0_	4.1	2.3	2.0	8.1
C_14:0_	1.0	TR	TR	TR
iso-C_15:0_	6.7	14.3	6.3	8.5
anteiso-C_15:0_	5.3	35.7	29.7	39.1
C_15:1_*ω6c*	2.2	TR	TR	TR
iso-C_16:1_ H	1.1	TR	1.5	3.7
iso-C_16:0_	30.0	14.6	18.3	16.9
SF 3^*a*^	8.9	TR	1.0	1.5
C_16:0_	9.1	2.0	7.2	3.6
SF 9^*b*^	3.2	2.1	1.0	1.2
anteiso-C_17:1_ *w9c*	TR	1.8	2.8	2.1
C_17:0_ iso	5.1	5.5	4.2	2.2
anteiso-C_17:0_	4.6	15.5	21.1	10.2
C_17:0_ cyclo	ND	1.0	1.0	ND
C_17:0_	3.9	TR	TR	TR
iso-C_18:0_	1.0	1.9	1.7	ND

*Strains: 1, strain P38-E01^*T*^; 2, S. *daliensis* DSM 42095^*T*^; 3, S. *rimosus* subsp. *rimosus* NRRL B-2659^*T*^; 4, S. *sclerotialus* NRRL B-2317^*T*^.*

*All data were obtained in this study.*

*Values of less than 1% are not shown.*

*^*a*^C_16:1_*ω7c*/C_16:1_*ω6c*); ^*b*^C_16:0_ 10-methyl/iso-C_17:1_*ω9c*; ND, not detected; TR, trace amounts.*

### Phylogenetic Analysis of Strain P38-E01^*T*^

**FIGURE 2:**
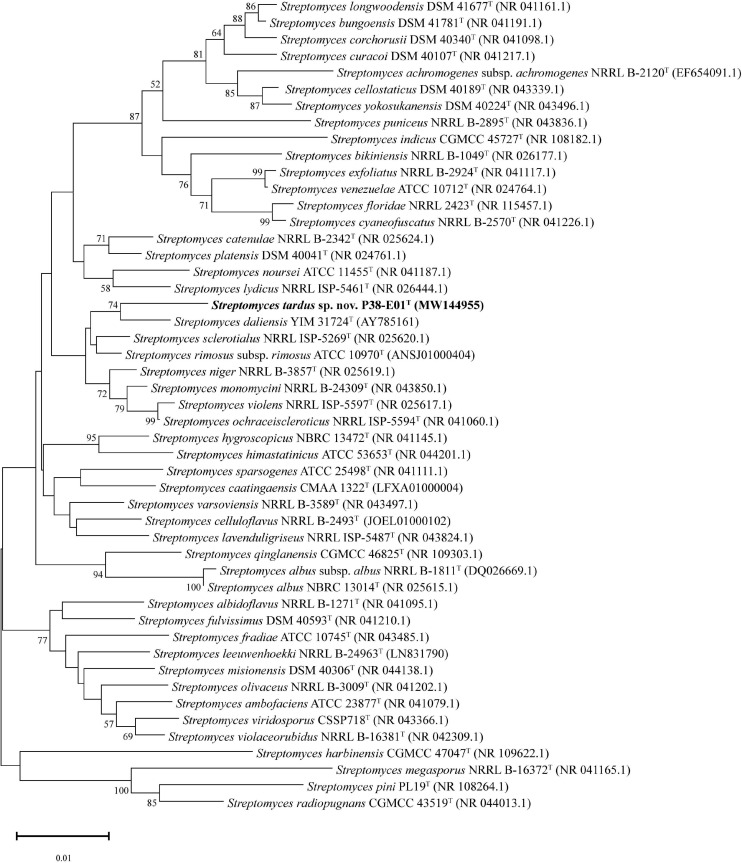
Neighbor-joining tree based on 16S rRNA gene sequences showing the relationship between strain P38-E01^*T*^ and related taxa. Only bootstrap values above 50% (percentages of 1,000 replications) are indicated. Bar, 0.01 nucleotide substitutions per site.

**FIGURE 3:**
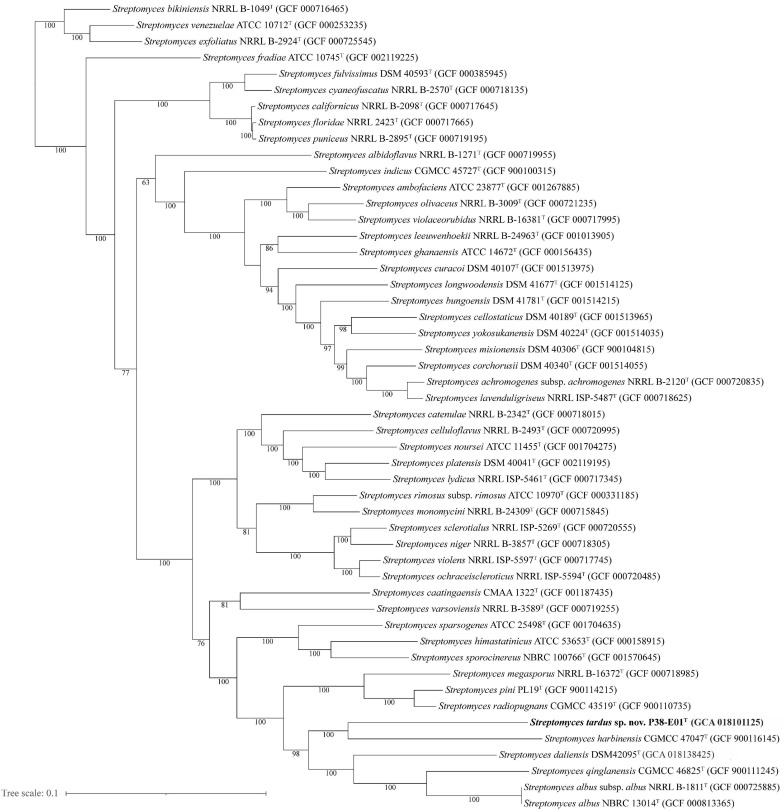
Maximum likelihood tree generated by alignment of 100 core proteins using autoMLST (Alanjary et al., 2019) showing the relationship between *S. tardus* strain P38-E01^*T*^ (shown in bold) and 50 of the closest related taxa. Bootstrap values based on 1,000 replications are depicted at nodes, and only values >50% are shown. Bar, 0.1 nucleotide substitutions per site.

The closest phylogenetic neighbors identified using the EzBioCloud database based on 16S rRNA gene sequences were *S. daliensis* YIM 31724^*T*^ (98.6%), *S. rimosus* subsp. *rimosus* ATCC 10970^*T*^ (98.4%), and *S. sclerotialus* NRRL ISP-5269^*T*^ (98.3%). Phylogenetic analysis of the 16S rRNA gene sequences revealed in the neighbor-joining phylogenetic tree (Figure 2) and the maximum-likelihood tree (Supplementary Figure 2) that P38-E01^*T*^ forms a cluster with *S. daliensis* YIM 31724^*T*^. In the maximum parsimony (Supplementary Figure 3) tree P38-E01^*T*^ formed an independent phylogenetic lineage close to *S. daliensis* YIM 31724^*T*^. MLSA analysis further supported that strain P38-E01^*T*^ belongs to the genus *Streptomyces* and formed a monophyletic group with *Streptomyces harbinensis* CGMCC 47047^*T*^ (GCF_900116145) (Figure 3). To define the phylogenetic position based on whole-genome sequences, a GBDP tree was calculated. Phylogenomics showed that strain P38-E01^*T*^ formed a distinct lineage within a monophyletic cluster comprising *Streptomyces alkaliterrae* OF1^*T*^ (GCA_007097205.2) and *Streptomyces durbertensis* DSM 104538^*T*^ (GCA_014156695.1) (Supplementary Figure 4). This cluster was further closely related to *S. harbinensis* CGMCC 47047^*T*^ and *S. daliensis* DSM 42095^*T*^ in concordance with the MLSA and 16S rRNA phylogenetic analysis.

The whole-genome sequencing showed that the genome of strain P38-E01^*T*^ consists of 6,158,643 bp assembled in 10 scaffolds with an average coverage of 364-fold. The DNA %G + C content was 71.4%, which is in concordance with the genus characteristics (Kämpfer, 2015). Further genomic information on strain P38-E01^*T*^ is listed in Table 3. ANI values between P38-E01^*T*^ and its closest relatives based on 16S rRNA, MLSA, and GBDP phylogenetic analyses ranged between 76.2 and 79.7% (Supplementary Figure 5). These values are well below the ANI threshold value (95–96%) established for delineation of novel species (Richter and Rosselló-Móra, 2009; Lee et al., 2016). DNA–DNA relatedness values between strain P38-E01^*T*^ and its closest phylogenetic relatives ranged between 21.6 and 24.1%(Supplementary Table 2), values below the cutoff value of 70% recommended for species delineation (Goris et al., 2007). Together, the genomic comparisons from ANI and GGDC further confirmed that strain P38-E01^*T*^ represents a novel species of the genus *Streptomyces*.

**TABLE 3:** Genomic characteristics of *S. tardus* P38-E01^*T*^.

	*S. tardus* sp. nov.
WGS Project no.	095364
Assembly method	*De novo* hybrid assembly workflow based on ABySS version 2.0.2, BLASR version 1.3.1, SSPACE-LongRead version 1.0, GapFiller version 1.10, and Pilon version 1.21
Genome size	6,158,643
Mean Coverage Illumina (×)	82
Mean Coverage PacBio (×)	364
N50	4,878,575
N75	4,878,575
L50	1
L75	1
Largest contig (bp)	4,878,575
No. of contigs > 200 bp	10
No. of contigs > 1,000 bp	7
No. of gaps	60
Total gaps size	6,943
GC content (%)	71.4
Coding sequences	5,071
No. of assigned COGs classes	21
No. of RNAs	80
No. of rRNAs	3
No. of tRNAs	62
No. of prophages	4 (incomplete)
No. of plasmids	0
CRISPRs	6

### Genome Annotation

**FIGURE 4:**
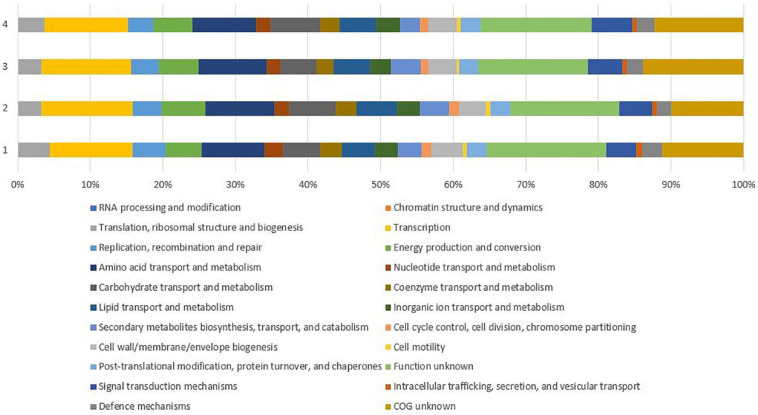
Comparison of gene functional categories of the strain P38-E01^*T*^ and the phylogenetically closest *Streptomyces* spp. identified in the 16S rRNA phylogenetic analysis. 1, strain P38-E01^*T*^; 2, *S. daliensis* DSM 42095^*T*^; 3, *S. rimosus* subsp. *rimosus* ATCC 10970^*T*^; 4, *S. sclerotialus* NRRL ISP-5269^*T*^.

Genome annotation revealed 5,151 genes further divided into 3,111 operons by Operon-mapper (Taboada et al., 2018), out of which 5,071 accounted for protein-coding genes (CDS), 80 for RNA genes including 3 rRNA genes (one cope of each, 5S, 16S, and 23S) and 62 tRNA genes. The prediction and classification of functional groups were based on evolutionary genealogy of genes resulting in Clusters of Orthologous Groups of proteins (COGs) (Huerta-Cepas et al., 2019). The vast majority of the predicted genes could not be assigned to any specific functional group (*n* = 529) and/or specific function (*n* = 774), together accounting for 25.69% of all CDSs. The remaining CDSs (*n* = 3,414) were assigned to specific COG classes (Supplementary Table 3), with most belonging to class (K) transcription (*n* = 538), class (E) amino acid transport and metabolism (*n* = 406), class (G) carbohydrate transport and metabolism (*n* = 240), followed by class (C) energy production and conversion (*n* = 235), class (I) lipid transport and metabolism (*n* = 212), and class (T) signal transduction mechanisms (*n* = 196). Comparison of CDSs between P38-E01^*T*^ and reference genomes showed similar distribution of CDSs into COG classes with most genes not assigned to any specific functional groups or to specific functions (Figure 4).

As one of the largest annotated groups was the COG class (G) carbohydrate transport and metabolism, the set of enzymes involved in the synthesis, breakdown, and transport of carbohydrates, the strain P38-E01^*T*^ genome contains 186 CAZyme domain sequences, with the most abundant groups identified as glycoside hydrolases (GHs, *n* = 63, 24 families) and glycosyl transferases (GTs, *n* = 60, 15 families). This finding suggests that strain P38-E01^*T*^ may be a source of industrially important enzymes, as GHs and GTs are frequently used in industrial applications such as juice and wine industries, animal feed industry, and textile industries (Raveendran et al., 2018). In addition, carbohydrate esterases (CEs, *n* = 14, 7 families), polysaccharide lyases (PLs, *n* = 1, 1 family), carbohydrate-binding molecules (CBMs, *n* = 25, 13 families), and redox enzymes assigned as auxiliary activities (AAs, *n* = 9) were also found. Interestingly, the only polysaccharide lyase detected in the genome of P38-E01^*T*^ belonged to the family PL31 and was described as an endo-β-1,4-glucuronan lyase with β-glucuronans as a specific substrate (Helbert et al., 2019). This suggests that strain P38-E01^*T*^ is able to degrade cellouronate, a salt from oxidized cellulose, which aligns with the phenotypic characteristics observed in this study from carbohydrate utilization tests. Furthermore, no enzymes supporting the ability to degrade pure cellulose were predicted within the genome, and this finding was supported by the lack of cellulose degradation by strain P38-E01^*T*^ when tested with a Whatman filter paper. Although functional prediction of COGs did not assign any genes to the class (X) Mobilome, phages and transposons, four incomplete prophage sequences, were detected on four different contigs, and Prophage Hunter assigned three of them as ambiguous regarding their potential activity (Supplementary Table 4). No plasmids were detected.

The *in silico* predicted resistome of strain P38-E01^*T*^ revealed discrepancies between the predicted antibiotic resistance and the phenotypically expressed resistance observed in this study. Using >50% sequence similarity and 100 ± 5% length cutoff values after comparison to known antibiotic resistance genes, 14 putative antibiotic resistance genes were found in P38-E01^*T*^ genome (Supplementary Table 5). These genes were found to encode resistance to 15 different antibiotic classes, including amphenicols, penicillins, and cephalosporines, which correlates with the findings that this strain is resistant to chloramphenicol (30 μg), penicillin G (10 μg), and cefixime (5 μg). Resistance to chloramphenicol is likely encoded by chloramphenicol phosphotransferase (*cmvl*) (GenBank accession number MBU7597020.1). The genome also encodes a sequence for a *soxR* gene of *P. aeruginosa* (GenBank accession number MBU7598185.1), a transcriptional factor that mediates protection against oxygen-radical generating toxic metals and antibiotics (Brown et al., 2003). The *soxR* gene appears highly conserved among *Streptomyces* spp. as a homolog to *P. aeruginosa soxR* gene, known to reduce susceptibility to various antibiotics including chloramphenicol (Koutsolioutsou et al., 2005), which may add to overall resistance of P38-E01^*T*^ against phenicol antibiotics. Resistance against β-lactams, including penams, cephalosporines, and carbapenems is widespread within Actinobacteria as it represents a self-defending mechanism against their own secondary metabolites with antibiotic activities (Ogawara, 2016). Major resistance mechanisms against β-lactams among *Streptomyces* spp. are the production of β-lactamases, penicillin-binding proteins (PBPs) and presence of PASTA domains (Ogawara, 2016). Interestingly, no genes encoding β-lactamases or PBPs were predicted in the P38-E01^*T*^ genome. Either these genes are yet unrecognized, hidden in categories of genes with unknown function, or this may be caused by a different mechanism, specifically by multidrug efflux mechanisms associated with *soxR* (GenBank accession number (MBU7598185.1) and *mtrA* (GenBank accession number MBU7598660.1). Based on the predicted resistome, resistance against fluoroquinolones, macrolides, tetracyclines, and aminoglycosides would be expected; however, phenotypically strain P38-E01^*T*^ was found to be susceptible against chosen antibiotics from these classes, which may be caused either by incorrect genome annotation, inactivity of putative resistance genes, or simply their suppression caused by the conditions chosen for antibiotic susceptibility testing.

Six putative CRISPRs are in the P38-E01^*T*^ genome (Supplementary Table 6). Three of these putative CRISPRs were predicted with high confidence (scoring 6.44–6.58), one with a medium confidence (score 4.65) and two with low confidence (scores 4.59 and 4.72). All, except for one high scoring CRISPRs, were predicted with a forward direction. All three high scoring CRISPRs were assigned to I-E type CRISP-Cas9 systems similarly as a CRISPR array discovered in *Streptomyces avermitilis* (Qiu et al., 2016). Seven CRISPR-associated proteins forming the so-called cascade (CRISPR-associated complex for anti-viral defense) (Brouns et al., 2008) were detected in the genome encoded by *casA*, *casB*, and *cas1*–cas7 genes. P38-E01^*T*^ also harbors *cas3*, a gene putatively encoding an essential protein in the I-E system with two functional domains (Westra et al., 2012) and the *cas7/cst2/devR* gene predicted to encode a CRISPR-associated negative autoregulator (Weiss and Sampson, 2014; see Supplementary Table 6). CRISPR-Cas technology is a powerful tool for editing genomes, especially valuable for genomic manipulation of genetically recalcitrant organisms. A novel knock-in CRISPR-based approach introducing *kasO^∗^p* promoter cassette to drive expression of putative BGCs was successfully used for *Streptomyces roseosporus*, *Streptomyces venezuelae*, and *Streptomyces viridochromogenes* (Zhang et al., 2017) leading to expression and production of novel secondary metabolites. Thus, the presence of putative CRISPR arrays, along with all known cascade proteins and *cas3* in P38-E01^*T*^ genome, implicates the activity of CRISPR/Cas immune system in strain P38-E01^*T*^; hence, future possibilities for activation of its silent and unusual BGCs using CRISPR-based tools.

### *In silico* Analysis of Secondary Metabolite Biosynthetic Gene Clusters

**FIGURE 5:**
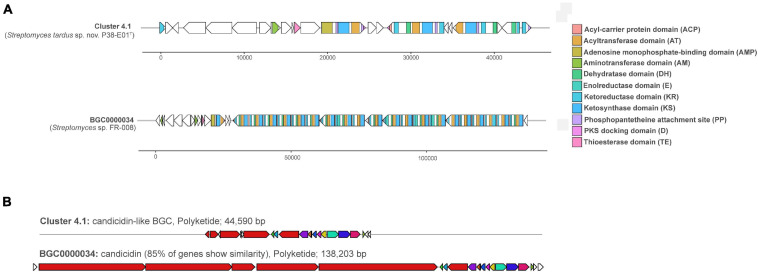
Annotation and similarity of a candicidin-like biosynthetic gene cluster (BGC) (BGC 9.1) and its analogous cluster (BGC0000034) predicted by antiSMASH (Blin et al., 2017) from strain P38-E01^*T*^. **(A)** Annotation of both clusters with ORFs are depicted as white arrows, and BGC domains are color coded. **(B)** Similarity of BGC 9.1 from strain P38-E01^*T*^ to a PKS candicidin cluster (BGC0000034) from *Streptomyces* sp. FR-008 (Chen et al., 2003).

Genome mining for potential secondary metabolite-associated biosynthetic gene clusters (BGCs) led to the identification of 19 clusters related to secondary metabolite biosynthesis as shown in Supplementary Table 7. Five of the clusters were predicted to match known BGCs encoding ectoine (Prabhu et al., 2004), staurosporine (Onaka et al., 2002), desferrioxamine (Sosio et al., 2018), geosmin (Redenbach et al., 1996), and alkylresorcinol (Ohnishi et al., 2008) with 100% similarity. Additional clusters matching known BGCs with high similarity included cluster 2.1, which was 95% similar to WS9326, a non-ribosomal peptide-encoding cluster from *Streptomyces calvus* ATCC 13382^*T*^, and cluster 4.1 with 85% similarity to a polyketide candicidin cluster (MIBiG accession # BGC0000034), which encodes the antifungal polyene metabolite, candicidin D1, produced by *Streptomyces* sp. FR-008 (Chen et al., 2003). Candicidin is an aromatic polyene macrolide antibiotic first described in *Streptomyces griseus* with potent activity against *Candida* species (Lechevalier et al., 1953), but has since been observed from other soil and marine *Streptomyces* species (Jørgensen et al., 2009). Previous studies have shown that the candicidin BGC is widely distributed in *Streptomyces* spp. isolated from sediments in the Trondheim fjord of Norway, and the genes involved in candicidin production were present on a linear plasmid suggesting that this BGC may be spread by conjugative transfer of the plasmid among *Streptomyces* spp. in their native habitat (Jørgensen et al., 2009). An annotation of the predicted candicidin-like cluster (Cluster 4.1) in strain P38-E01^*T*^ and the closest match BGC0000034 from *Streptomyces* sp. FR-008 is depicted in Figure 5A. Differences observed in the annotation of these candicidin-encoding clusters (Figure 5B) may be due to fragmentation of the BGC from genome assembly of strain P38-E01^*T*^, since the putative candicidin-like cluster (44,590 bp) is much smaller than that of BGC0000034 (138,203 bp).

The remaining BGCs that lacked significant matches (<70% identity) were predicted to encode an array of secondary metabolite families, most notably type I polyketide synthases (PKSs), non-ribosomal peptide synthetases (NRPSs), ribosomally synthetized and post-translationally modified peptides (RiPPs), and lassopeptides. Specifically, three BGCs showed no similarity to any reference gene clusters. In total, 2 out of 31 BGCs were predicted to be associated with BGCs with antitumor activities, but the vast majority of identified BGCs showed similarities to clusters associated with antimicrobial activities. The core structure was predicted for 11 out of 19 clusters, including mostly NRPS, PKS type I, NRPS-like, and PKS–NRPS-like hybrid clusters. Additionally, two core peptides were also predicted for two putative lassopeptides.

### Antimicrobial Activity of Strain P38-E01^*T*^

The results from the antibacterial assays (Supplementary Figure 6) demonstrated that supernatants collected from strain P38-E01^*T*^ were capable of inhibiting the growth of the Gram-positive MRSA strains Xen29 and EAMC30, *M. luteus* ATCC 10240, and the bacterial plant pathogen strains *C. michiganensis* subsp. *michiganensis* 89C-4 and *C. flaccumfaciens* subsp. *flaccumfaciens* CV3 when cultured in YEME broth for 7 days. The prediction of BGCs that could be responsible for the biosynthesis of antifungal metabolites by antiSMASH analysis (Supplementary Table 6) was further supported by the antimicrobial assays, which demonstrated that supernatants from strain P38-E01^*T*^ inhibited the growth of the fungal strain *C. albicans* ATCC 90028 when grown in YEME broth for 7 days with an observed ZOI of 9 mm. Antifungal activity was lost when strain P38-E01^*T*^ was cultured for more than 14 days (Supplementary Figure 6) indicating that the antifungal metabolites produced may have degraded over time. The greatest degree of antibacterial activity, determined by the size of each zone of inhibition, was observed against *C. michiganensis* subsp. *michiganensis* 89C-4 (14 mm ZOI). No activity was observed against any test organism when P38-E01^*T*^ was cultured in GYE, MF, TSB, or ISP 2 broth. Additionally, no activity against Gram-negative strains was observed when cultured in any of the media types tested, suggesting that the produced metabolites by strain P38-E01^*T*^ specifically act against Gram-positive and/or fungal strains.

**FIGURE 6:**
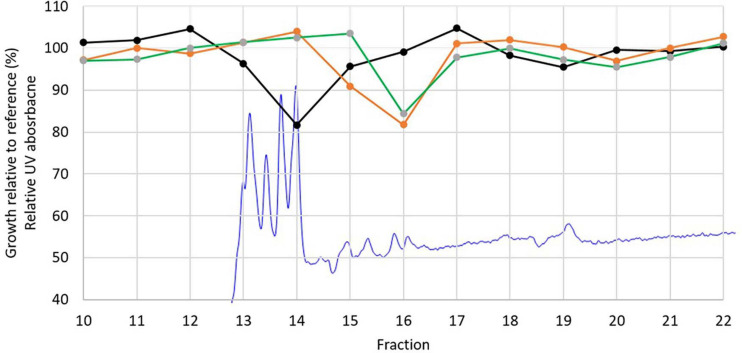
Relative UV absorption chromatogram at 270 nm from the fractionation of DMSO extract of P08-E01^*T*^ (blue) overlayed with the growth inhibition results of *Candida albicans* CCUG 39333 (black), *Enterococcus faecium* CCUG 37832 (orange), and *Kocuria rhizophila* ATCC 9341 (green).

Cultivation of strain P38-E01^*T*^ in three media followed by bioassay showed that the highest bioactivity was obtained against *C. albicans* CCUG 39333 in extracts prepared from 5334SW and 5254SW cultures. These extracts resulted in 35–40% growth relative to the control when a 16× diluted DMSO extract was added. To identify the compounds responsible for the observed bioactivity, extracts from the 5254SW culture of strain P38-E01^*T*^ were fractionated using HPLC connected to a DAD detector and a fraction collector, and the fractions were tested for activity against *C. albicans* CCUG 39333, *K. rhizophila* ATCC 9341, *and E. faecium CCUG 37832*. Activity was found in two fractions (fractions 14 and 16) both showing growth reduced to approximately 80% relative to the reference. Fraction 14 inhibited *C. albicans*, whereas fraction 16 inhibited *E. faecium* and *M. luteus*. Four characteristic UV peaks were observed in fraction 14 (Figure 6).

**FIGURE 7:**
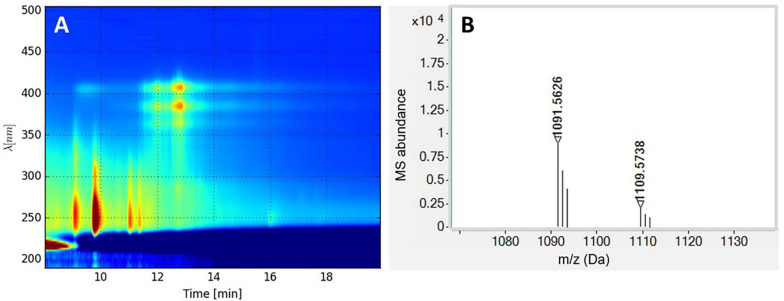
**(A)** LC-diode array detector (DAD) isoplot of the analyzed extract showing candicidin at retention time 13–14 min. **(B)** MS spectrum (M + H) of candicidin and candicidin-H_2_O (right).

The identity of the four main peaks eluting in fraction 14 (13–14 min in Figure 6) was investigated by LC-DAD-QTOF of the extract (Figure 7). The neutral mass of the first peak was *M* = 1,108.5695, which corresponds to candicidin with the molecular formula C_59_H_84_N_2_O_18_. The mass of peaks 2 and 3 both corresponded to a dehydration derivative or MS adduct of candicidin with neutral mass *M* = 1,090.5580. UV data of the fourth peak strongly indicate that this peak is candicidin or a candicidin derivative. The identity of the compound(s) inhibiting growth of *K. rhizophila* and *E. faecium* in fraction 16 could not be determined from these analyses. Taken together, the prediction of a candicidin-like cluster by antiSMASH, the MS identification of candicidin, and the activity against *C. albicans* suggest that the antifungal activity from strain P38-E01^*T*^ is linked to its ability to produce a candicidin antibiotic.

## Conclusion

In summary, the phenotypic, chemotaxonomic, and genomic analyses performed in this study confirmed that strain P38-E01^*T*^ belongs to the genus *Streptomyces* and represents a novel species. Colony morphology and growth were comparable with characteristics of members of the genus *Streptomyces*. Fatty acid profile as well as other chemotaxonomic characteristics further confirmed features typical for the genus *Streptomyces.* Strain P38-E01^*T*^ possessed unique morphological and biochemical features that can be used for its identification and differentiation from its closest phylogenetic neighbors analyzed in this study. Phylogenetic analyses indicated a separate lineage of strain P38-E01^*T*^ within the genus *Streptomyces*, and comparative genomics calculating low orthoANI values as well as low dDDH values further confirmed the genomic distance of strain P38-E01^*T*^ from closely related reference strains. Additionally, the strain P38-E01^*T*^ genome harbors a significant number of biosynthetic genes with one-half showing little to no similarity to known biosynthetic clusters, suggesting considerable potential of this strain to produce diverse bioactive secondary metabolites. This is also supported by our findings that strain P38-E01^*T*^ inhibits growth of plant pathogens and Gram-positive pathogens including MRSA strains and fungi. Although the antifungal activity was linked to the previously described compound candicidin, other antimicrobial compounds responsible for antibiotic activity are yet to be identified. Moreover, the presence of seven CRISPR/*cas* genes in the P38-E01^*T*^ genome indicates that manipulation of the P38-E01^*T*^ genome with a CRISPR/Cas system may facilitate future studies of the biological significance of specific metabolites encoded by P38-E01^*T*^ BGCs. In summary, our results showed not only biosynthetic potential of strain P38-E01^*T*^ for biopharmaceutical field but also its distinguishable phenotypic and genotypic properties confirming that it represents a novel species of the genus *Streptomyces* for which the name *Streptomyces tardus* sp. nov. is proposed.

### Description of *Streptomyces tardus* sp. nov.

*Streptomyces tardus* (tar’dus. L. masc. adj. *tardus* slow, pertaining to the slow growth).

This is a Gram-stain positive actinobacterium that forms branched substrate mycelium with long filaments on ISP 2 medium. Each branch produces smooth spores at the end and grows well on ISP 2, 4, 5, 6, 7, nutrient agar, and modified Bennett’s agar. No growth is observed on ISP 3 and Czapek–Dox agar. This is a slow-growing bacterium with growth observed no earlier than after 14 days. Growth occurs at 28–32°C with optimum growth at 30°C, pH 6.5–11, and 0–5.0% (w/v) NaCl. Melanoid pigment is not produced. Nitrates are not reduced to nitrites. Urease is not produced. D-fructose, D-galactose, D-glucose, D-mannitol, D-mannose, D-ribose, D-xylose, glycerol, and L-arabinose are utilized as carbon sources. D-lactose D-sorbitol, L-rhamnose, myo-inositol, raffinose, salicin, and sucrose are not utilized. It degrades DNA, gelatin, Tween 80, hypoxanthin, and xanthin. It is positive for casein, L-tyrosine, and starch hydrolysis and negative for esculin hydrolysis. Utilization of cellulose is positive; degradation of cellulose is negative. In the API ZYM, it is positive for acid phosphatase, alkaline phosphatase, α-glucosidase, α-mannosidase, β-galactosidase, β-glucosidase (weak), cysteine arylamidase (weak), esterase (C 4), esterase lipase (C 8), leucine arylamidase, lipase (C14), N-acetyl-β-glucosaminidase, napthol-AS-Bi-phosphohydrolase, and valine arylamidase. Negative tests with API ZYM are α-chymotrypsin, α-fucosidase α-galactosidase, β-glucuronidase, and trypsin. It is resistant to chloramphenicol (30 μg), penicillin G (10 μg), and cefixime (5 μg).

The diagnostic diamino acid of the peptidoglycan is LL-diaminopimelic acid. The whole-cell hydrolysate contains glucose, ribose, and minor amounts of mannose. The main polar lipids are diphosphatidylglycerol, phosphatidylethanolamine, phosphatidylglycerol, and phosphoglycolipid. The predominant menaquinones are MK-10(H_6_), MK-10(H_4_), and MK9(H_4_). The predominant fatty acids are iso-C_16:0_, C_16:0_, and Summed Feature 3.

The type strain, P38-E01^*T*^ (= CCM 9049^*T*^ = DSM 111582^*T*^), was isolated from sediments of the Trondheim fjord, Norway. The DNA %G + C content of the draft genome sequence (6.16 Mbp) of the type strain indicates a value of approximately 71.4 mol% for the species. The genome is predicted to contain 31 biosynthetic gene clusters encoding known and unknown secondary metabolites, and the isolate shows biological activity against *C. albicans* ATCC 90028 and the Gram-positive human and plant pathogens. The GenBank accession number for the assembled draft genome of *S. tardus* sp. nov. is JAELVF020000000.

## Data Availability Statement

The datasets presented in this study can be found in online repositories. The names of the repository/repositories and accession number(s) can be found below: https://www.ncbi.nlm.nih.gov/genbank/, MW144955, https://www.ncbi.nlm.nih.gov/genbank/, JAELVF020000000.

## Author Contributions

SK, MS-P, and DF, under the supervision of ML, performed all the microbiological strain characterizations, including morphology, growth conditions, biochemical and physiological characterization, antibiotic susceptibility, analysis of fatty acid methyl esters, and the antimicrobial activity tests, as well as genomic and phylogenetic analyses. GK was involved in the Actinobacteria strain isolation campaign and the maintenance of isolates at SINTEF, while AL performed strain cultivation, verification, and maintenance for this study. G-SN performed the bioinformatics analyses of the genome sequences and the metabolic potential of strains involved in this study. KD performed the LC-MS-based identification of bioactive compounds. AW supervised the genome sequencing by BaseClear, the analysis of peptidoglycan, quinones, and polar lipids at DSMZ, and, together with ML, acquired the funding for this study. SK drafted the manuscript, with inputs from MS-P, ML, KD, and G-SN, and finalized it together with ML and AW. All authors edited the draft manuscript and agreed to the final manuscript version for submission.

## Conflict of Interest

The authors declare that the research was conducted in the absence of any commercial or financial relationships that could be construed as a potential conflict of interest.

## Publisher’s Note

All claims expressed in this article are solely those of the authors and do not necessarily represent those of their affiliated organizations, or those of the publisher, the editors and the reviewers. Any product that may be evaluated in this article, or claim that may be made by its manufacturer, is not guaranteed or endorsed by the publisher.

## Footnotes

1
https://sourceforge.net/projects/bbmap/
