# Supplementary material for: Streptomyces tardus sp. nov.: A Slow-Growing Actinobacterium Producing Candicidin, Isolated From Sediments of the Trondheim Fjord
Source: Front Microbiol. 2021 Aug 4;12:714233. doi: 10.3389/fmicb.2021.714233 (PMC8371330; doi:10.3389/fmicb.2021.714233)
Supplement: Supplementary file 1 [file Data_Sheet_1.PDF]

# - Supplementary Material -

Streptomyces tardus sp. nov., a slow-growing actinobacterium producing candicidin A isolated from sediments of the Trondheim fjord.

Stanislava Králová [1,2], Megan Sandoval-Powers [1], Dorelle V. Fawwal [1], Kristin F. Degnes [3], Anna Sofia Lewin[3], Geir Klinkenberg [3], Giang-Son Nguyen [3], Mark R. Liles[1] and Alexander Wentzel [3]*

[1]Department of Biological Sciences, Auburn University, Auburn, AL, 36849, USA
[2]Department of Experimental Biology, Czech Collection of Microorganisms, Faculty of Science, Masaryk University,Brno, 62500, Czech Republic
[3]Department of Biotechnology and Nanomedicine, SINTEF Industry, 7034 Trondheim, Norway

*** Correspondence:** Alexander Wentzel. Address: Richard Birkelands vei 3, 7034 Trondheim, Norway. Phone: +4793200776. Email: alexander.wentzel@sintef.no

Table S1. Growth and cultural characteristics of strain P38-E01^{T} and type strains of the closest related *Streptomyces* spp.

Figure S1. Scanning electron micrograph of strain P38-E01^{T}.

Figure S2. Maximum-likelihood tree based on 16S rRNA gene sequences showing the relationship between strain P38-E01^{T} and related taxa. Only bootstrap values above 50 % (percentages of 1000 replications) are indicated. Bar, 0.01 nucleotide substitutions per site.

Figure S3. Maximum parsimony tree based on 16S rRNA gene sequences showing the relationship between strain P38-E01^{T} and related taxa. Only bootstrap values above 50 % (percentages of 1000 replications) are indicated. Bar, 20nucleotide substitutions per site.

Figure S4. Phylogenomic tree inferred with FastME 2.1.6.1 (Lefort et al., 2015) from GBDP distances calculated from genome sequences. Branch lengths are scaled in terms of GBDP distance formula d_5. Numbers above branches indicate GBDP pseudo-bootstrap support values from 100 replications with the average branch support of 95.7%.

Figure S5. OrthoANI heat map generated with OAT software showing ANI values calculated between strain P38-E01^{T} and type strains of the closest related *Streptomyces* spp.

Table S2. Digital DNA-DNA hybridization values calculated between strain P38-E01^{T} and type strains of the closestrelated Streptomyces spp.

Table S3. Clusters of orthologous groups of strain P38-E01^{T}.

Table S4. Putative prophages predicted by PHASTER and Phage Hunter.

Table S5. Antibiotic resistance genes harboured in P38-E01^{T} genome.

Table S6. CRISPR spacers and CRISPR-associated genes encoded in P38-E01^{T} genome.

Table S7. Secondary metabolite BGCs in the genome of strain P38-E01^{T} predicted with antiSMASH 4.2.0.

Figure S6. Antimicrobial activity of supernatants of strain P38-E01^{T}.

Table S1. Growth and cultural characteristics of strain P38-E01^T and type strains of the closest related *Streptomyces* spp.
Strains: 1, P38-E01^T; 2, S. *daliensis* DSM 42095^T; 3, S. *rimosus* subsp. *rimosus* NRRL B-2659^T; 4, S. *sclerotialus* NRRL B-2317^T. All data were obtained during this study.

**Medium**	**1**	**2**	**3**	**4**
ISP 2				
Aerial mycelium	Pale yellow	White/yellow	Yellow	Yellow
Substrate mycelium	Pale yellow	Yellow	Brown	Yellow
ISP 3				
Aerial mycelium	No growth	Light yellow	White	White
Substrate mycelium	No growth	Light yellow	White	White
ISP 4				
Aerial mycelium	White	Pale yellow with white borders	White	Pale yellow
Substrate mycelium	White	Pale yellow	Brown	Pale yellow
ISP 5				
Aerial mycelium	White	White/yellow	White	Yellow/white
Substrate mycelium	White	White	Pale yellow	Yellow
ISP 6				
Aerial mycelium	White	Light brown	Light brown	Yellow
Substrate mycelium	Yellow	Dark yellow	Brown	Yellow
ISP 7				
Aerial mycelium	White	Light brown	White	Yellow/brown/white
Substrate mycelium	Pale yellow	Light brown	Yellow/brown	Dark brown
Nutrient agar				
Aerial mycelium	White	Light brown, brown pigment	Yellow	Pale yellow
Substrate mycelium	Pale yellow	Light brown, brown pigment	Yellow	Pale yellow
Czapek's Dox agar				
Aerial mycelium	No growth	White	White	White
Substrate mycelium	No growth	White	White	White
Modified Bennett's agar				
Aerial mycelium	White	Dark beige	Yellow and white	Yellow
Substrate mycelium	Pale yellow	Dark beige	Light brown	Yellow

Figure S1. Scanning electron micrograph of strain P38-E01^{T}.

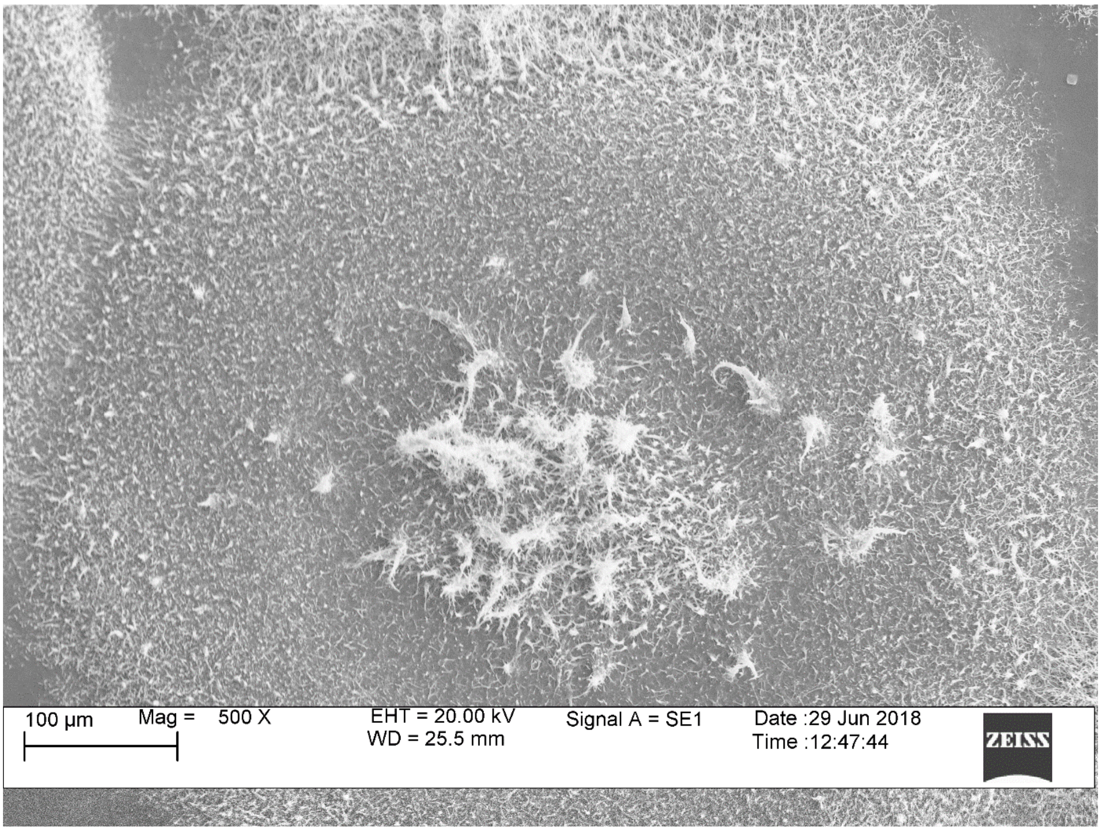

Figure S2. Maximum-likelihood tree based on 16S rRNA gene sequences showing the relationship between strain P38-E01^T and related taxa. Only bootstrap values above 50 % (percentages of 1000 replications) are indicated. Bar, 0.01 nucleotide substitutions per site.

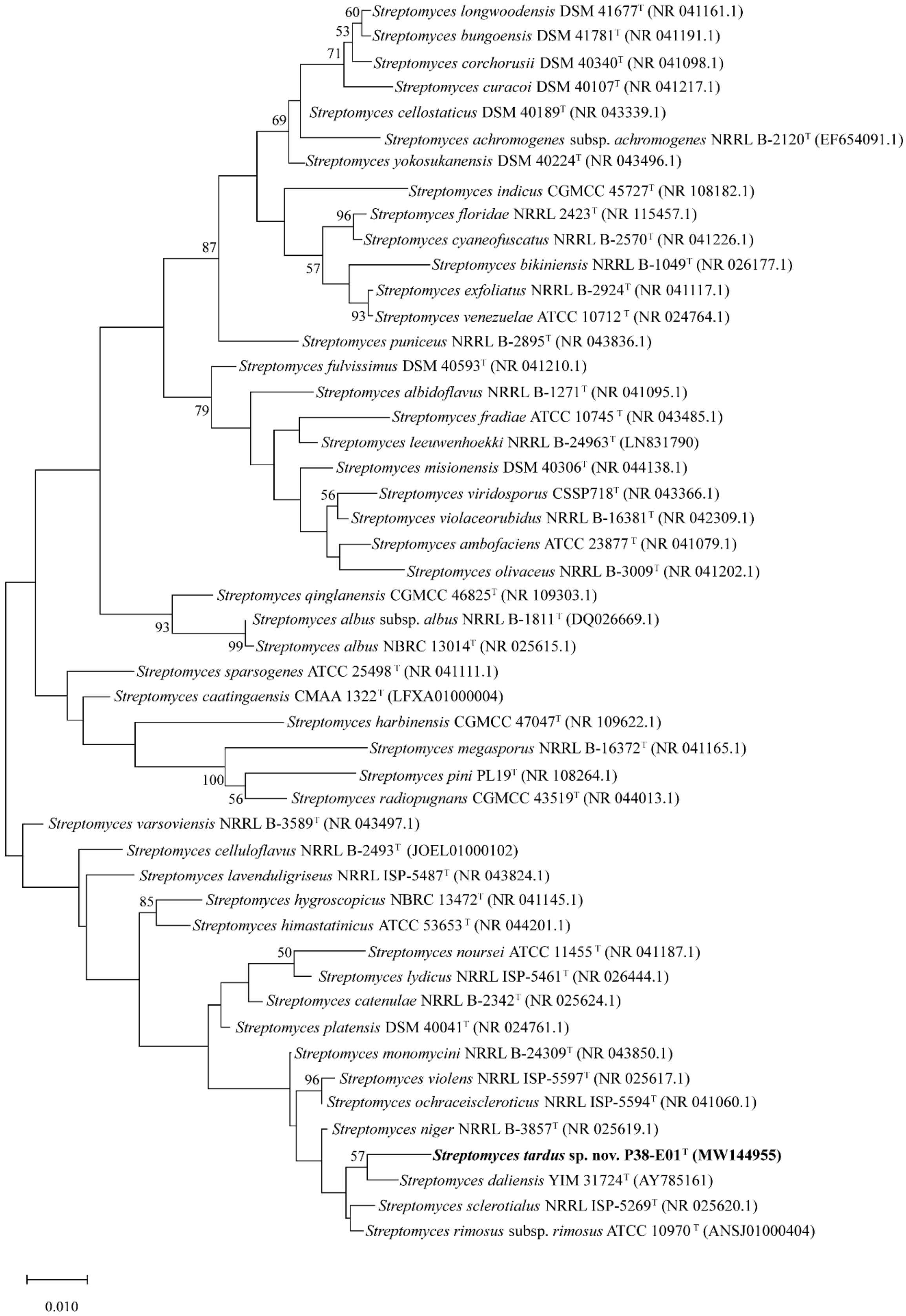

Figure S3. Maximum-parsimony tree based on 16S rRNA gene sequences showing the relationship between strain P38-E01^{T} and related taxa. Only bootstrap values above 50 % (percentages of 1000 replications) are indicated. Bar, 5 nucleotide substitutions per site.

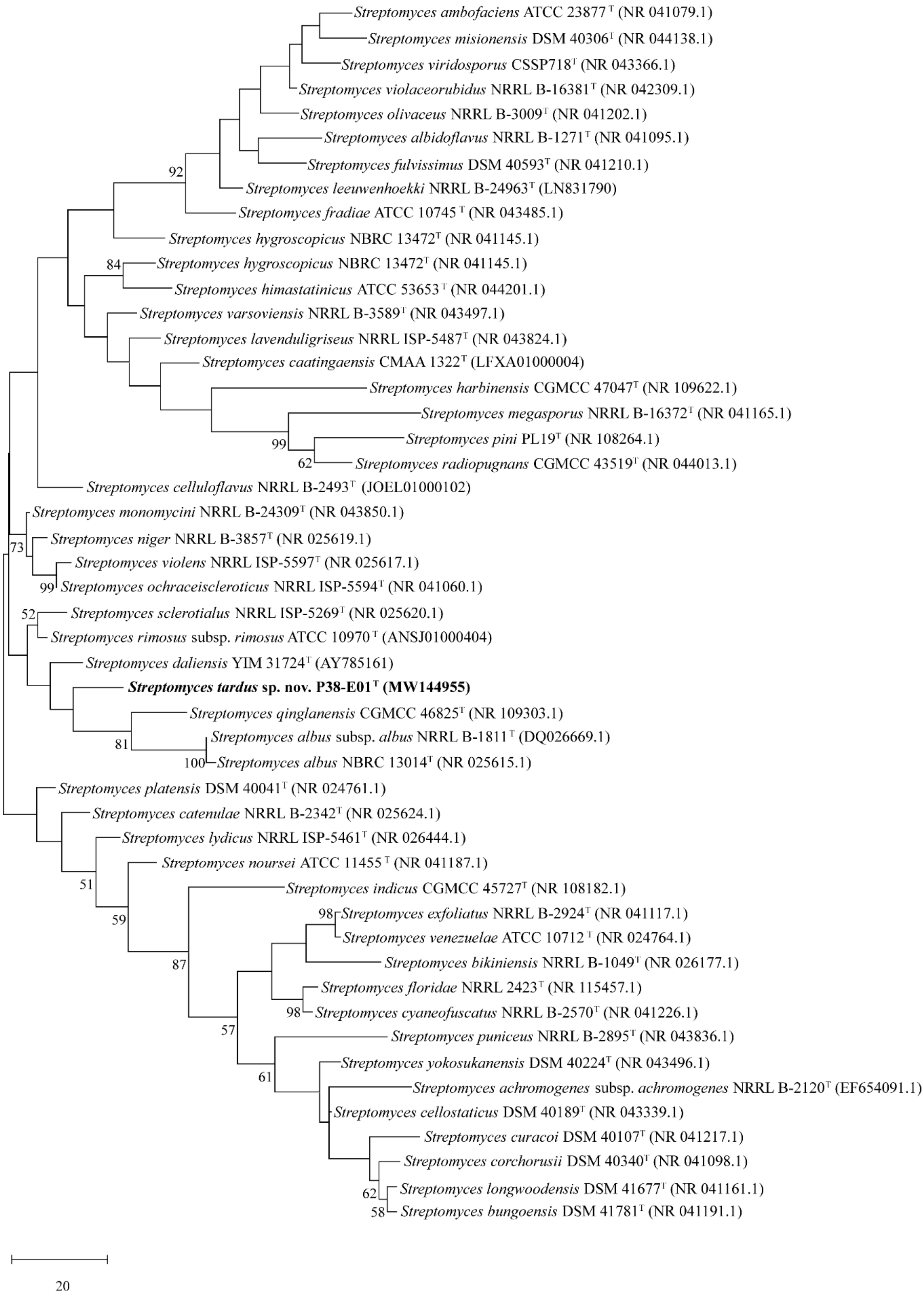

Figure S4. Phylogenomic tree inferred with FastME 2.1.6.1 (Lefort et al., 2015) from GBDP distances calculated from genome sequences. Branch lengths are scaled in terms of GBDP distance formula d_5. Numbers above branches indicate GBDP pseudo-bootstrap support values from 100 replications with the average branch support of 95.7%.

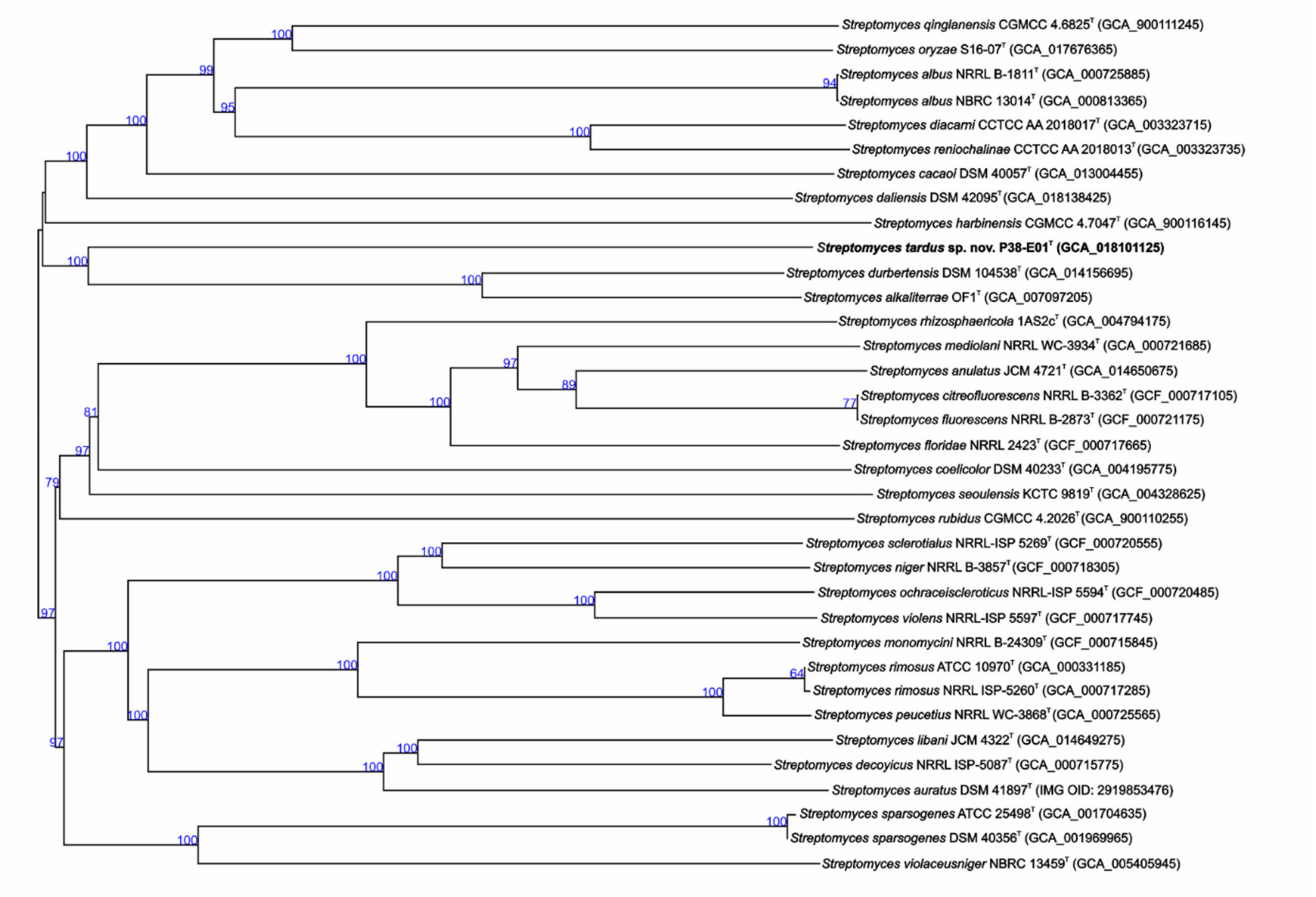

Figure S5. OrthoANI heat map generated with OAT software (Lee *et al.,* 2015) showing ANI values calculated between strain P38-E01^{T} and type strains of the closest related *Streptomyces* spp.

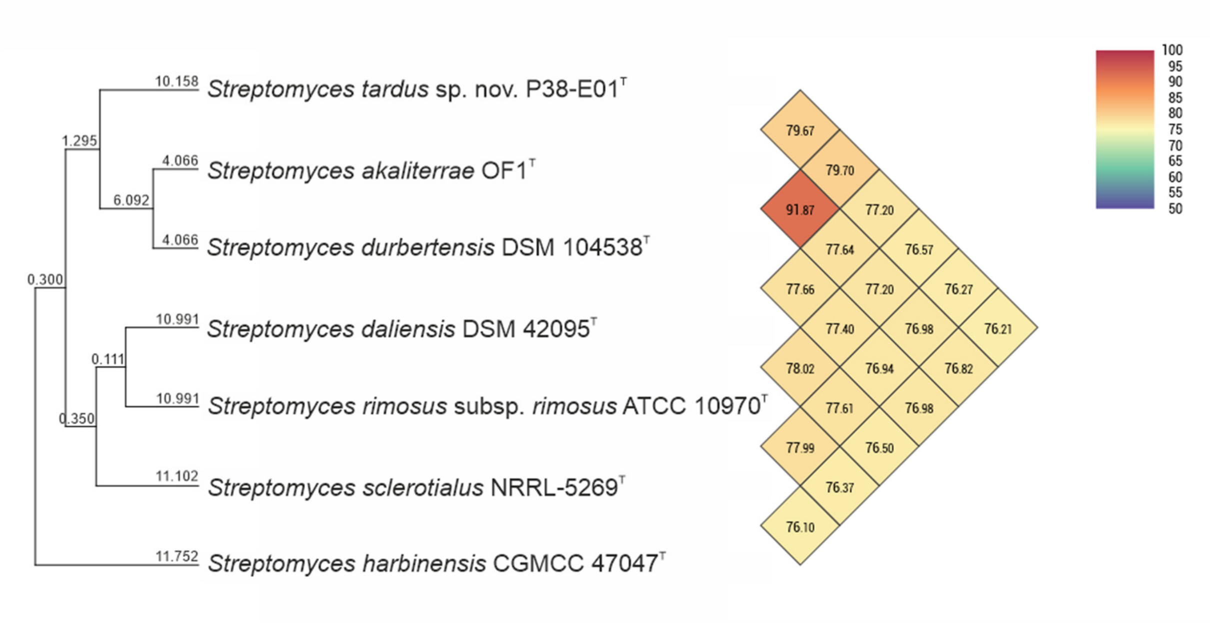

Table S2. Digital DNA-DNA hybridization values calculated between strain P38-E01^{T} and type strains of the closest related *Streptomyces* spp.

Type strains	P38-E01^{T}
S. tardus P38-E01^{T}	100 %
S. *daliensis* DSM 42095^{T}	22.6%
S. *rimosus* subsp. *rimosus* ATCC 10970^{T}	22.3%
S. *sclerotialus* NRRL ISP-5269^{T}	21.8%
S. harbinensis CGMCC 47047^{T}	21.8%
S. alkaliterrae OF1^{T}	24.1%
S. durbertensis DSM 104538^{T}	24.1%

Table S3. Clusters of orthologous groups of strain P38-E01^T.

COG class	Description	Gene count	Percentage
	**Information storage and processing**		
A	RNA processing and modification	3	0.06 %
B	Chromatin structure and dynamics	3	0.06 %
J	Translation, ribosomal structure and biogenesis	204	4.02 %
K	Transcription	538	10.61 %
L	Replication, recombination and repair	215	4.24 %
	**Metabolism**		
C	Energy production and conversion	235	4.63 %
E	Amino acid transport and metabolism	406	8.01 %
F	Nucleotide transport and metabolism	121	2.40 %
G	Carbohydrate transport and metabolism	240	4.73 %
H	Coenzyme transport and metabolism	141	2.78 %
I	Lipid transport and metabolism	212	4.18 %
P	Inorganic ion transport and metabolism	153	3.02 %
Q	Secondary metabolites biosynthesis, transport, and catabolism	152	3.02 %
	**Cellular processes and signalling**		
D	Cell cycle control, cell division, chromosome partitioning	64	1.26 %
M	Cell wall/membrane/envelope biogenesis	205	4.04 %
N	Cell motility	25	0.49 %
O	Post-translational modification, protein turnover, and chaperones	131	2.58 %
S	Function unknown	774	15.26 %
T	Signal transduction mechanisms	196	3.86 %
U	Intracellular trafficking, secretion, and vesicular transport	38	0.75 %
V	Defence mechanisms	132	2.60 %
	**Unknown category**		
	COG unknown	529	10.43 %

Table S4. Putative prophages predicted by PHASTER and Phage Hunter.

PHASTER

ID	Scaffold	Start	End	Category	No. of genes
P1	1	932139	940381	Incomplete*	8

Phage Hunter

ID	Scaffold	Start	End	Category	No. of genes
P2	1	1397483	1414307	ambiguous[¥]	8
P3	1	1906286	1921277	ambiguous	7
P4	1	3107517	3119617	ambiguous	9

* PHASTER categories: intact/questionable/incomplete [¥]Prophage Hunter categories: active/ambiguous/inactive

Table S5. Antibiotic resistance genes harboured in P38-E01^{T} genome.

Protein Accession Number	Best hit to Antibiotic Resistance Ontology (ARO)	ARO	Sequence similarity (%)	AMR gene family	Drug class	Resistance mechanism
MBU7596918.1	*Mycobacterium tuberculosis rpoB mutants* conferring resistance to rifampicin	3003283	74.64	rifampicin-resistant beta-subunit of RNA polymerase rpoB	rifampicin antibiotic	antibiotic target alteration antibiotic target replacement
MBU7598660.1	*mtrA*	3003369	74.11	resistance-nodulation-celldivision (RND) antibiotic efflux pump	macrolide antibiotic, penam	antibiotic efflux
MBU7596912.1	*Escherichia coli* EF-Tu mutants conferring resistance to Pulvomycin	3003369	73.67	elfamycin resistant EF-Tu	elfamycin antibiotic	antibiotic target alteration
MBU7597620.1	*Streptomyces lividans cmlR*	3002690	72.32	major facilitator superfamily (MFS) antibiotic efflux pump	phenicol antibiotic	antibiotic efflux
MBU7597914.1	*APH(3')-Ia*	3003638	67.45	APH(3')	aminoglycoside antibiotic	antibiotic inactivation
MBU7596918.1	*Mycobacterium tuberculosis gyrA* conferring resistance to fluoroquinolones	3003295	65.78	fluoroquinolone resistant gyrA	fluoroquinolone antibiotic	antibiotic target alteration
MBU7597020.1	*cmvl*	3002700	63.83	chloramphenicol phosphotransferase	phenicol antibiotic	antibiotic inactivation
MBU7597914.1	*APH(3')-IIa*	3002644	61.11	APH(3')	aminoglycoside antibiotic	antibiotic inactivation
MBU7599913.1	*iri*	3002884	60.00	rifampin monooxygenase	rifampin antibiotic	antibiotic inactivation
MBU7598185.1	*Pseudomonas aeruginosa soxR*	3004107	58.39	ATP-binding cassette (ABC) antibiotic efflux pump; major facilitator superfamily (MFS) antibiotic efflux pump; resistance-nodulation-cell division (RND) antibiotic efflux pump	fluoroquinolone antibiotic; cephalosporin; glycylcycline; penam; tetracycline antibiotic; acridine dye; rifamycin antibiotic; phenicol antibiotic; triclosan	antibiotic target alteration; antibiotic efflux
MBU7598762.1	*AAC(6´)-Iag*	3004638	57.14	AAC(6´)	aminoglycoside antibiotic	antibiotic inactivation
MBU7597544.1	*rtgt1438*	3002883	54.95	rifampin glycosyltransferase	rifampin antibiotic	antibiotic inactivation
MBU7598599.1	*oleB*	3003036	53.10	ABC-F ATP-binding cassette ribosomal protection protein	macrolide antibiotic; lincosamide antibiotic; streptogramin antibiotic; tetracycline antibiotic; oxazolidinone antibiotic; phenicol antibiotic; pleuromutilin antibiotic	antibiotic targetprotection
MBU7598173.1	*mphO*	3004543	52.17	macrolide phosphotransferase (MPH)	macrolide antibiotic	antibiotic inactivation

Table S6. CRISPR spacers and CRISPR-associated genes encoded in P38-E01^T genome.

CRISPR arrays

CRISPR ID	Scaffold	Start position	End position	Number of repetitions	Repeat sequence	Repeat length	Spacer length	Direction	Array family	Confidence score	
CRISPR 1	151	239733	240689	15	GTCGTCATCAGCCCTGGAGGGCTCGCAAC	29	37	F	NA	4.59	low
CRISPR 2	122	251978	253990	31	CCCGTCATCAGCCCTGGAGGGCTCGCAAC	29	37	F	NA	4.65	medium
CRISPR 3	122	26260	265462	42	GTCCTCATCAGCCCTGGAGGGCTCGCAAC	29	37	F	NA	4.72	low
CRISPR 4	126	3539787	3538659	19	GTGCTCTCCGCGCGAGCGGAGGTGAACCGC	30	31	F	I-E	6.58	high
CRISPR 5	152	3550968	3552339	23	GTGCTCTCTGCGCGAGCGGAGGTGAACCGC	30	31	R	I-E	6.44	high
CRISPR 6	333	3553673	355595	27	GTGCTCTCTGCGCGAGCGGAGGTGAACCGG	28	35	F	I-E	6.47	high

Gene	Protein accession number	Length (bp)	Family and superfamily	Description	CRISPR type
casA	MBU7598911.1	1580	NA	CRISPR-associated protein Cse1/CasA	I-E
casB	MBU7598912.1	746	NA	CRISPR-associated protein Cse2/CasB	I-E
cas1b	MBU7600234.1	980	COG1518	CRISPR/Cas system-associated endonuclease Cas1	I-B
cas1e	MBU7598916.1	917	COG1518	CRISPR/Cas system-associated endonuclease Cas1	I-E
cas2	MBU7600233.1	263	COG3512	CRISPR/Cas system-associated endonuclease Cas2	NA
cas2e	MBU7598917.1	311	COG3512	CRISPR/Cas system-associated endoribonuclease Cas2	I-E
cas3	MBU7598910.1	2836	COG1203	CRISPR-associated endonuclease Cas3	I
cas3	MBU7600236.1	2426	COG1203	CRISPR-associated helicase Cas3	I
cas4	MBU7600235.1	512	COG1468	CRISPR-associated protein Cas4	I, II-B
cas5	MBU7600237.1	677	COG1688 (RAMP)*	CRISPR-associated protein Cas5	NA
cas5	MBU7598914.1	833	COG1688 (RAMP)	CRISPR-associated protein Cas5	I-E
cas6	MBU7600240.1	689	(RAMP)	CRISPR system Cascade subunit CasE	NA
cas6/cse3/casE	MBU7598915.1	668	(RAMP)	CRISPR/Cas system-associated protein Cas6/Cse3/CasE	I-E
cas7e/ces4/casC	MBU7598913.1	1226	(RAMP)	CRISPR/Cas system-associated protein Cas7/Cse4/CasC	I-E
cas7/cst2/devR	MBU7600238.1	1058	COG1857 (RAMP)	CRISPR-associated autoregulator DevR	I-B
TIGR03984 family CRISPR associated gene	MBU7600230.1	617	TIGR03984 (RAMP)	CRISPR associated protein	NA
TIGR03986 family CRISPR associated gene	MBU7600231.1	2507	TIGR03986 (RAMP)	CRISPR associated protein	NA

*RAMP (Repair Associated Mysterious Proteins) – identified proteins with unknown functions, NA - information not provided.

Table S7. Secondary metabolite BGCs in the genome of strain P38-E01T predicted with antiSMASH 6.0 (Blin et al., 2021). CDPS, tRNA-dependent cyclodipeptide synthesis; hglE-KS, heterocyst glycolipid synthase-like PKS; NRPS, non-ribosomal peptide synthetase; T1 PKS, Type I polyketide synthase; T3 PKS, Type III polyketide synthase.

Cluster type	MIBiG BGC-ID	Most similar known cluster	Identity (%)	Size (bp)	Effects
arylpolyene, lanthipeptide class II, NRPS, T3PKS	BGC0000282	Alkylresorcinol	100	127,782	-
arylpolyene, NRPS, NRPS-like, ladderane	BGC0001297	WS9326	95	85,565	-
NRPS-like	BGC0000893	Chloramphenicol	52	42,817	antibacterial [1]
terpene	BGC0001181	Geosmin	100	22,322	-
T1PKS	BGC0000163	Tetronasin	3	57,918	-
hglE-KS	BGC0000935	Esmeraldin	8	51,870	antitumor [2]
CDPS	-	-	-	20,729	-
thiopeptide, lantipeptide	BGC0002028	Frigocyclinone	20	28,956	antibacterial, antitumor [3]
lassopeptide	-	Putative macrolactame	-	22,552	likely antimicrobial
ectoine	BGC0000853	Ectoine	100	10,405	stress-protectant [4]
siderophore	BGC0001478	Desferrioxamine E	100	11,806	ion-chelation [5]
nucleoside	-	-	-	20,366	-
lassopeptide	BGC0001551	Citrulassin E	66	28,738	antibacterial
NRPS-like, T1PKS	BGC0000034	Candicidin	85	44,590	antifungal [6]
lassopeptide	BGC0001550	Citrulassin D	40	24,872	antibacterial
siderophore	BGC0000244	Macrotetrolide	33	16,774	-
NRPS, T1PKS, indole	BGC0000825	Staurosporine	100	61,625	antibacterial [7]
T1PKS	BGC0001491	67-121C	29	102,711	antifungal [8]
T1PKS	BGC0000035	Chalcomycin A	4	12,077	antibacterial [9]

Figure S6. Antimicrobial activity of supernatants of strain P38-E01^{T} against select organisms by a spot-on-lawn assay.

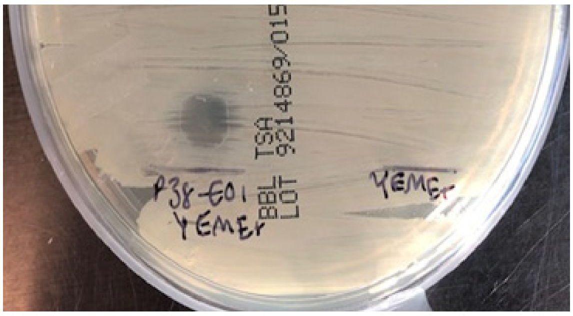

Curtobacterium flaccumfaciens subsp. *flaccumfaciens* CV3

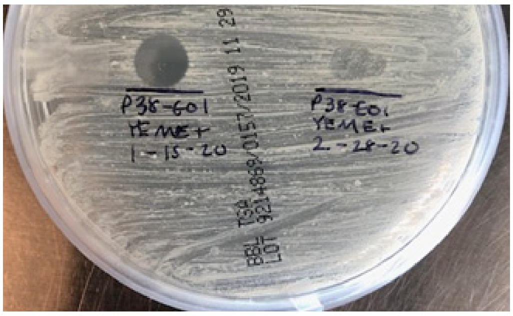

Candida albicans ATCC 90028

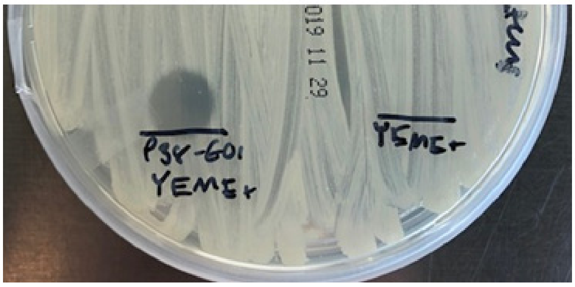

Micrococcus luteus ATCC 10240

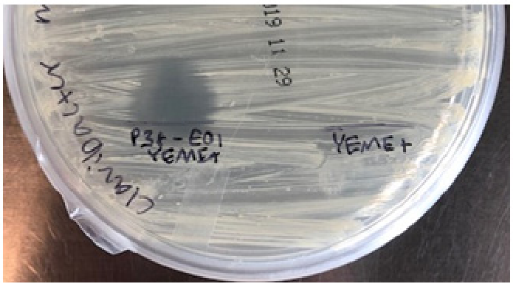

Clavibacter michiganensis subsp. *michiganensis* 89C-4

References:

[1] Fernández-Martínez LT, Borsetto C, Gomez-Escribano JP, Bibb MJ, Al-Bassam MM, Chandra G, et al. New Insights into Chloramphenicol Biosynthesis in *Streptomyces venezuelae* ATCC 10712. *Antimicrob Agents Chemother* 2014;58:7441–50. https://doi.org/10.1128/AAC.04272-14.

[2] Rui Z, Ye M, Wang S, Fujikawa K, Akerele B, Aung M, et al. Insights into a Divergent Phenazine Biosynthetic Pathway Governed by a Plasmid-Born Esmeraldin Gene Cluster. *Chemistry & Biology* 2012;19:1116–25. https://doi.org/10.1016/j.chembiol.2012.07.025.

[3] Mo J, Ye J, Chen H, Hou B, Wu H, Zhang H. Cloning and identification of the Frigocyclinone biosynthetic gene cluster from *Streptomyces griseus* strain NTK 97. *Bioscience, Biotechnology, and Biochemistry* 2019;83:2082–9. https://doi.org/10.1080/09168451.2019.1638755.

[4] Richter AA, Mais C-N, Czech L, Geyer K, Hoeppner A, Smits SHJ, et al. Biosynthesis of the Stress-Protectant and Chemical Chaperon Ectoine: Biochemistry of the Transaminase EctB. *Front Microbiol* 2019;10. https://doi.org/10.3389/fmicb.2019.02811.

[5] Yamanaka K, Oikawa H, Ogawa H, Hosono K, Shinmachi F, Takano H, et al. Desferrioxamine E produced by *Streptomyces griseus* stimulates growth and development of Streptomyces tanashiensis. *Microbiology* n.d.;151:2899–905. https://doi.org/10.1099/mic.0.28139-0.

[6] Jørgensen H, Fjærvik E, Hakvåg S, Bruheim P, Bredholt H, Klinkenberg G, et al. Candicidin Biosynthesis Gene Cluster Is Widely Distributed among *Streptomyces* spp. Isolated from the Sediments and the Neuston Layer of the Trondheim Fjord, Norway. *Appl Environ Microbiol* 2009;75:3296–303. https://doi.org/10.1128/AEM.02730-08.

[7] Onaka H, Taniguchi S, Igarashi Y, Furumai T. Cloning of the staurosporine biosynthetic gene cluster from *Streptomyces* sp. TP-A0274 and its heterologous expression in *Streptomyces lividans. J Antibiot* (Tokyo) 2002;55:1063–71. https://doi.org/10.7164/antibiotics.55.1063.

[8] Sheehan J, Murphy CD, Caffrey P. New insights into polyene macrolide biosynthesis in *Couchioplanes caeruleus*. *Mol BioSyst* 2017;13:866–73. https://doi.org/10.1039/C7MB00112F.

[9] Coffey GL, Anderson LE, Douros JD, Jr ALE, Fisher MW, Hans RJ, et al. CHALCOMYCIN, A NEW ANTIBIOTIC: BIOLOGICAL STUDIES. *Canadian Journal of Microbiology* 2011. https://doi.org/10.1139/m63-088.
